# Quantum refinement can be used to improve time-resolved crystallographic structures

**DOI:** 10.1107/S2052252526009486

**Published:** 2026-11-05

**Authors:** Gayathri Yuvaraj, Kristoffer J. M. Lundgren, Esko Oksanen, Ulf Ryde

**Affiliations:** a Division of Computational Chemistry Lund University Chemical Centre, PO Box 124 221 00Lund Sweden; b European Spallation Source ESS ERIC PO Box 176 221 00Lund Sweden; c LINXS – Institute of Advanced Neutron and X-ray Science, Mesongatan 4, 224 84Lund, Sweden; King’s College London, United Kingdom; University of Padova, Italy

**Keywords:** time-resolved crystallography, quantum refinement, bacteriorhodopsin, xylose isomerase, fluoroacetate dehalogenase

## Abstract

Time-resolved serial crystallography has been shown to be a powerful technique to study enzyme reactions. In such studies, a reaction is triggered within the crystal and data are collected at different timepoints. A problem with this approach is that the synchronization is never perfect and therefore the structures at different timepoints typically contain a mixture of states in the active site, for example involving unreacted states, intermediates and products. Interpreting the electron density of mixtures is a challenge for crystallographic modelling and the resulting structures depend strongly on the quality of the empirical restraints employed. In this study, we investigate the potential of using quantum refinement for time-resolved structures, *i.e.* replacing the empirical restraints with more accurate quantum-mechanical calculations for the active site. We studied three different proteins. The first is a structure of bacterio­rhodopsin containing a mixture of Schiff bases of all-*trans* and 13-*cis* retinal (PDB entry 5b6w). We show that quantum refinement gives similar structures to the original study, but the strain energies (indicating how much the structures are distorted from the optimum geometry) are much smaller: 36 and 71 kJ mol^−1^ for the all-*trans* and 13-*cis* conformations, respectively, compared with 366 and 473 kJ mol^−1^ for the deposited structure. Next, we studied a reaction intermediate of xylose isomerase (PDB entry 6qni), showing that the structure probably contains a mixture of the substrate-free state, as well as both the ring-closed and ring-opened glucose substrate, in contrast to the deposited structure, which was interpreted as 100% ring-closed glucose. Third, we studied a structure of fluoroacetate dehalogenase (PDB entry 6qhp), which was interpreted as a mixture of a covalent reaction intermediate and two conformations of the fluoroacetate substrate. Our best interpretation (with much less difference density) contains no intermediate and only a single conformation of the substrate, as well as a water molecule. We also studied all of the other structures in the time-resolved study of this enzyme to show that quantum refinement gives consistent structures throughout the reaction. We show that the suggestion of two conformations of the intermediate in the other chain of the protein is also questionable and point out that it is hard to discriminate between the fluoro­acetate substrate and the glycolate product. For all structures, we also perform standard crystallographic refinement, with empirical restraints instead of the quantum-mechanical calculations. These often give similar results, but since electrostatic interactions are ignored they cannot properly describe hydrogen bonds, which are key to provide reliable positions of water molecules and other polar groups, especially in the poorly defined electron density of mixed states in time-resolved data.

## Abstract 2

We show that quantum refinement can be used to improve the interpretation of structures from time-resolved serial crystallography. Examples are shown for bacteriorhodopsin, xylose isomerase and fluoroacetate dehalogenase, leading to better interpretations and improved structures in terms of difference maps, real-space *Z*-scores and strain energies.

## 1. Introduction

Accurate atomistic structures are the basis for understanding the function and mechanisms of biological macromolecules such as proteins and nucleic acids. They also open the possibility of rationally manipulating their function by mutations or the development of drug candidates. X-ray crystallography has long been the prime source of structural information on biological macromolecules (Rhodes, 2006 ▸), but during the latest decade, cryogenic electron microscopy has become a competitive complement (Cheng *et al.*, 2015 ▸). In most X-ray crystallographic studies, the structural information has been limited to single structures of equilibrium states, such as apo states, unreactive substrates and inhibitors.

However, the development of powerful X-ray sources, such as synchrotrons and X-ray free-electron lasers, now provides the opportunity to collect series of structures for dynamic events or reactions in a time-resolved manner (Schmidt, 2019 ▸; Pearson & Mehrabi, 2020 ▸; Orville, 2020 ▸; Barends *et al.*, 2022 ▸). An experimental challenge is to synchronize the unit cells in the crystal to the same state in the dynamic event (Hekstra, 2023 ▸). This is normally ensured by starting the event by a laser pulse, triggering the reaction by a photochemical event, a temperature or pH jump, or diffusing in the substrate (Pearson & Mehrabi, 2020 ▸; Brändén & Neutze, 2021 ▸; Pandey *et al.*, 2020 ▸).

A second challenge is to interpret the resulting data. Since the triggering of the dynamic event is seldom fully efficient and synchronized, the collected data typically reflect a mixture of structures, representing unreacted states as well as various dynamic states (De Zitter *et al.*, 2022 ▸). For example, data from a chemical reaction may involve a mixture of the reactant, the product and possible intermediate states. Consequently, the resulting electron density will be a mixture of all of these states and it is a challenge to sort out what is really seen, especially at the limited resolution typically obtained for biomacromolecules (Srinivasa Raghavan *et al.*, 2025 ▸). Normally, the structures are interpreted with standard crystallographic methods, assuming mixtures of several states with non-unity occupancies and using empirical restraints to ensure that the resulting structures make chemical sense (Afonine *et al.*, 2012 ▸). Therefore, the results strongly depend on the accuracy of the restraints employed.

Quantum refinement (QR) is a variant of crystallographic refinement in which the restraints are replaced by quantum-mechanical (QM) calculations for a small but interesting part of the structure (Ryde *et al.*, 2002 ▸; Bergmann *et al.*, 2022 ▸). This improves the description of groups that are poorly determined by standard restraints, for example cofactors, substrates, inhibitors, drug candidates, metal sites and water molecules. It also provides a more accurate description of the site of interest and the closest surroundings by using QM calculations. Restraints in crystallographic software (and molecular-mechanics force fields in general) are often extracted from QM calculations, but they employ a simple model system, assuming that all interactions of a similar type have the same behaviour (Moriarty *et al.*, 2009 ▸; Vanommeslaeghe *et al.*, 2014 ▸). Moreover, they use a simplified description of the interaction (typically as a second-order Taylor expansion) and ignore electrostatic interactions. These approximations are avoided by QR.

QR has been used to locally improve X-ray crystal structures, to determine protonation states of metal-bound ligands, to determine oxidation states of metals and other groups, to detect photoreduction and to discriminate between different interpretations of a structure (Ryde & Nilsson, 2003 ▸; Nilsson & Ryde, 2004 ▸; Cao *et al.*, 2017 ▸; Bergmann *et al.*, 2021*a* ▸,*b* ▸; Borbulevych *et al.*, 2021 ▸; Cao *et al.*, 2020 ▸; Yuvaraj *et al.*, 2025 ▸). It has been extended to other methods, such as NMR, extended X-ray absorption fine structure, neutron crystallo­graphy, cryogenic electron microscopy, X-ray free-electron laser data and electron crystallography (Hsiao *et al.*, 2005 ▸, 2006 ▸; Caldararu *et al.*, 2019 ▸; Lundgren *et al.*, 2024 ▸, 2026 ▸). Moreover, it has been implemented to describe systems with multiple conformations in the QM region, which is key to the description of mixtures in time-resolved structures (Cao & Ryde, 2020 ▸; Lundgren *et al.*, 2024 ▸). In addition, it has been implemented in the widely used macromolecular structure-determination software *Phenix* (Lundgren *et al.*, 2024 ▸; Liebschner *et al.*, 2019 ▸). Other groups have implemented the same or related approaches, for example treating the whole macromolecule with QM or with QM-fitted machine-learning potentials (Bergmann *et al.*, 2022 ▸; Yu *et al.*, 2005 ▸; Hsiao *et al.*, 2010 ▸; Fadel *et al.*, 2015 ▸; Zheng *et al.*, 2017 ▸, 2020 ▸; Yan *et al.*, 2021 ▸; Wang *et al.*, 2020 ▸, 2023 ▸; Zubatyuk *et al.*, 2025 ▸).

In this study, we examine the potential of QR to interpret time-resolved X-ray crystallographic data and evaluate electron densities that reflect mixtures of states. When the electron density is ambiguous, the exact location of atoms in the structure will mainly be determined by the empirical restraints, and QM calculations can be expected to give a more accurate and potentially less biased interpretation of the density compared with parametrized restraints. Moreover, QR is ideal to study chemical reaction intermediates and other structures that are hard to describe with standard restraints, for example metal sites (Hu & Ryde, 2011 ▸). Here, we study three systems, bacteriorhodopsin, xylose isomerase and fluoroacetate dehalogenase, employing time-resolved data from previous investigations (Nango *et al.*, 2016 ▸; Mehrabi, Schulz, Agthe *et al.*, 2019 ▸; Mehrabi, Schulz, Dsouza *et al.*, 2019 ▸).

## 2. Methods

In standard crystallographic refinement, a target function of the following type is optimized,
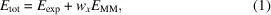
where *E*_exp_ is the experimental (crystallographic) target function, *E*_MM_ is the standard crystallographic restraints for the entire system (an MM function in terms of computational chemistry) and *w_x_* is a weight factor to measure the relative importance of the *E*_exp_ and *E*_MM_ target functions. In quantum refinement, this target function is extended in the following way
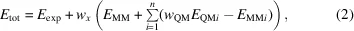
where the sum is over *n* different conformations of the QM region, *E*_QM*i*_ is the QM energy of the *i*th conformation of the QM region and *E*_MM*i*_ are the crystallographic restraints for the same region. Finally, *w*_QM_ is another weight factor that is needed because *E*_QM*i*_ is in energy units, whereas *E*_MM*i*_ and *E*_MM_ are in arbitrary (statistical) units (Lundgren *et al.*, 2024 ▸). Thus, a separate QM calculation of the complete QM region is performed for each conformation. All quantum refinements were performed with the *QRef* interface (Lundgren *et al.*, 2024 ▸) between the *Phenix* crystallographic software (version 1.21.2-5419; Liebschner *et al.*, 2019 ▸) and the *ORCA* QM software (version 5.0.4; Neese *et al.*, 2020 ▸). The *QRef* interface can be downloaded from https://github.com/krlun/QRef, where installation and usage instructions are also available.

The refinements were based on the following PDB structures: PDB entries 5b6w (Nango *et al.*, 2016 ▸), 6qni (Mehrabi, Schulz, Agthe *et al.*, 2019 ▸), 6gxh, 6qhy, 6qhv, 6qhu, 6qht, 6qhs, 6gxd, 6qhq, 6gxt, 6qhp, 6qhw, 6qhx, 6qhz, 6qi0, 6qi1, 6qi2 and 6qi3 (Mehrabi, Schulz, Dsouza *et al.*, 2019 ▸). Coordinates, occupancies, atomic displacement parameters (ADPs) and reflection data were downloaded from the PDB (Berman *et al.*, 2000 ▸), together with the space group, unit-cell parameters, resolution limits, *R* values and the test set used for the evaluation of the *R*_free_ values. For bacteriorhodopsin, the r5b6wsf data set was used.

H atoms for all atoms were included in the refinements and the other calculations for bacteriorhodopsin because they were present in the deposited PDB file. For the other two proteins, H atoms were included only in the QM region in the quantum refinements (because they are required in the QM calculations). They were added for xylose isomerase and fluoroacetate dehalogenase using *phenix.ready_set*. The protonation state is quite clear for all residues in the QM regions (shown in the figures), except His54 in xylose isomerase, for which we tested two protonation states (discussed below). In the quantum refinements, H atoms are individually refined, meaning that their positions are almost entirely determined by the QM calculations. Restraints for the nonstandard residues were generated using *phenix.elbow* (Moriarty *et al.*, 2009 ▸).

Reciprocal-space refinement was performed with *phenix.refine* (Afonine *et al.*, 2012 ▸), involving three macrocycles, where each macrocycle consisted of coordinate refinement, followed by refinement of the individual ADPs (isotropic in all three studied proteins). In the coordinate refinement, only coordinates of residues with at least one atom in the QM region were allowed to move. In the ADP refinement, ADPs for all atoms were allowed to change. After that, real-space *Z-*scores based on the difference maps (RSZDs) were calculated by the use of *EDSTATS* (Tickle, 2012 ▸), employing coordinates from the refinement and maps generated by *phenix.maps* (these maps were also used for all figures). Maps and RSZD values were calculated in the same way from deposited structures without performing any refinement. Reported RSZD values are for the entire residues. The *Phenix* software was also used in the original time-resolved studies of our three test cases.

To pinpoint the effect of QR on the structures, we performed also standard crystallographic refinement (SCR; *i.e.* without QM calculations) on all structures treated with QR. The two sets of refinements were performed with exactly the same settings so that the results reflect only the difference between the use of QM or the *Phenix* empirical restraints for the residues in the QM region. Thereby, we also study the effect of using updated versions of the *Phenix* software and the empirical restraints in the refinement.

All QM calculations were performed with the TPSS density-functional theory method (Tao *et al.*, 2003 ▸) and the def2-SV(P) basis set (Schäfer *et al.*, 1992 ▸). We employed the DFT-D4 dispersion correction (Caldeweyher *et al.*, 2019 ▸). The QM regions were selected following standard best practices in QM-cluster and QM/MM calculations (Neese, 2006 ▸; Senn & Thiel, 2009 ▸; Blomberg *et al.*, 2014 ▸; Ryde, 2016 ▸) and are described for the various proteins below (and are shown in the figures). The *w*_*x*_ scale factor in equations (1) and (2) was determined as the average of the values estimated in the two last macrocycles of a *Phenix* refinement without any QM, as was suggested in our previous QR studies (Lundgren *et al.*, 2024 ▸; Yuvaraj *et al.*, 2025 ▸). It was 1.80 for bacteriorhodopsin, 3.33 for xylose isomerase and 5.00 for fluoroacetate dehalogenase. It was kept fixed in all QR calculations to ensure that results with different interpretations of the structure are comparable. The *w*_QM_ weight factor in equation (2) was kept at 7.5 mol kcal^−1^, as was recommended in our previous study (Lundgren *et al.*, 2024 ▸). Detailed information on how to set up the QR calculations can be found at https://signe.teokem.lu.se/ulf/Methods/qref.html. Xylose isomerase contains two metal ions: magnesium and cobalt. They were modelled in the Mg^2+^ and high-spin Co^2+^ states, respectively.

Strain energies were calculated by comparing the QM energies of the QM region for the QR structures and the corresponding structure obtained by a full geometry optimization of the isolated QM region using the same QM method, keeping the atoms that are converted from C to H atoms (to truncate the QM region; the CD atom of Lys216 in bacterio­rhodopsin, the CA atom of Asp110 for fluoroacetate dehalogenase and nine CA or CB atoms for xylose isomerase) fixed in their original locations to ensure that residues do not move to unrealistic positions (Lundgren *et al.*, 2024 ▸). Strain energies for ligands bound to proteins have been much discussed and the consensus is that they are low for neutral noncovalent ligands when evaluated with proper solvation (Boström *et al.*, 1998 ▸; Sitzmann *et al.*, 2012 ▸; Peach *et al.*, 2017 ▸). For larger regions that are covalently connected to the protein, evaluation of the strain energies becomes more complicated and system-dependent (Ryde, 2002 ▸; Lundgren *et al.*, 2024 ▸), but the fact that restraint and QM terms are included in the target function in equation (1) or (2) that should be minimized shows that crystallographic refinement aims at minimizing strain energies.

All occupancy refinements were performed with *phenix.refine* using the same occupancy groups as in the deposited structure or in the corresponding QR structures (two to four groups). The occupancies were always constrained to sum to unity. Further details of the occupancy refinements are given below for the specific proteins.

A typical convergence of the quantum-refinement process is shown in Supplementary Fig. S1. Even if *R*_free_ may change by 0.007 between the third and tenth macrocycle, strain energies change by less than 1 kJ mol^−1^ and RSZD scores by no more than 0.1, showing that the discussed results are properly converged.

## 3. Results and discussion

### 3.1. Bacteriorhodopsin

We started by studying bacteriorhodopsin. This protein uses the energy of light to drive proton transport, building up a transmembrane proton gradient that is used to form ATP. It contains a retinal molecule, connected by a protonated Schiff base to a lysine residue (Lys216), which traps light, converting from the all-*trans* state to the 13-*cis* state, and triggering a conformational change in the protein. In 2016, Iwata and coworkers published a time-resolved X-ray crystallographic study of bacteriorhodopsin, triggering the reaction with a nanosecond laser pulse and collecting 14 structures from 16 ns to 1.725 ms after triggering at room temperature to a resolution of 2.1 Å (Nango *et al.*, 2016 ▸). We have concentrated on the structure obtained 16 ns after photoexcitation (PDB entry 5b6w), because it contains the K state in which the *cis*-retinal is in a twisted conformation that can be expected to be hard to describe using standard molecular-mechanics (MM) restraints.

**Figure 1:**
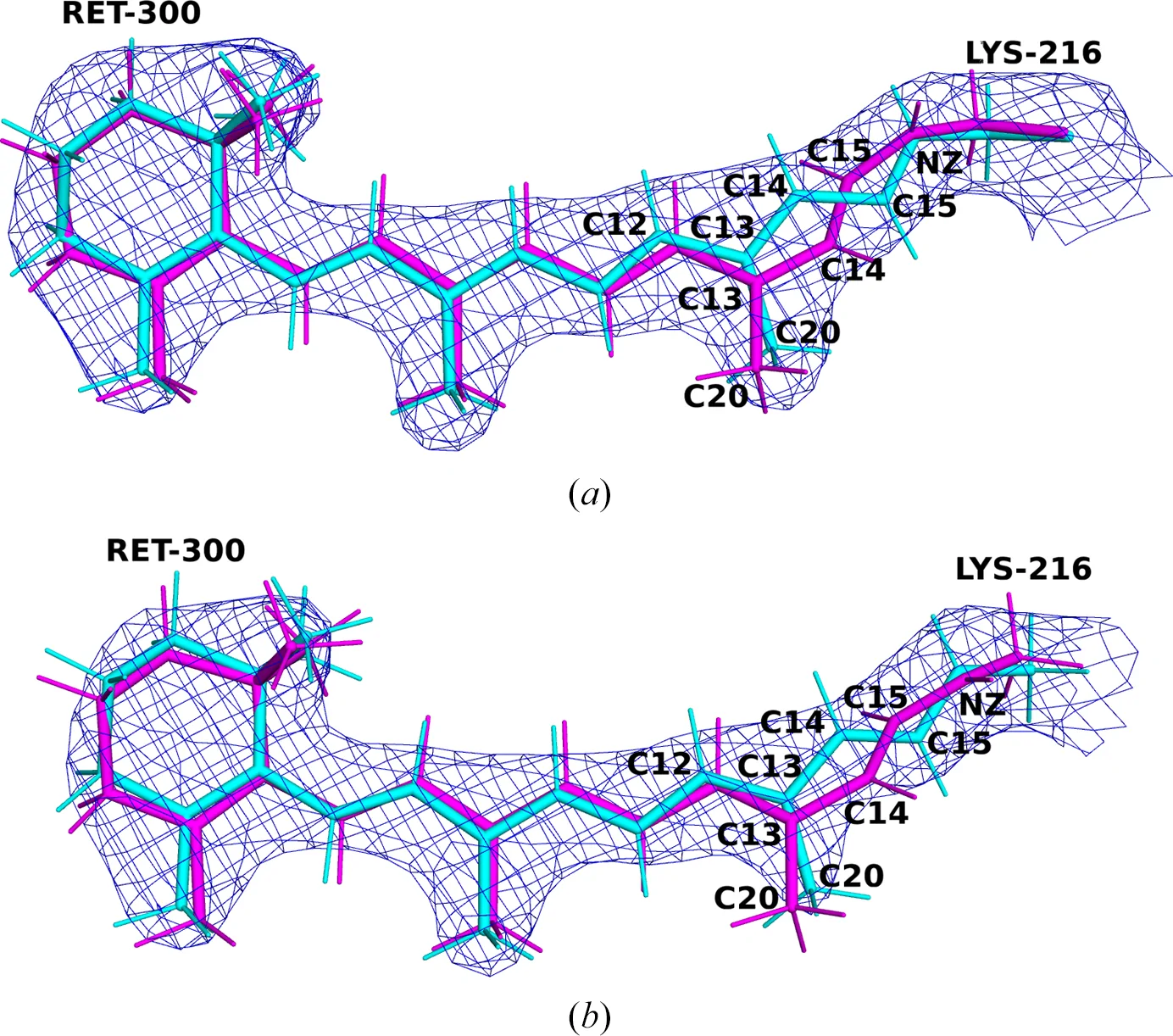
(*a*) Deposited structure of bacteriorhodopsin (PDB entry 5b6w). (*b*) QR structure with 85% occupancy for the all-*trans* state (cyan) and 15% occupancy for the 13-*cis* state (magenta). This figure also shows the QM region used in the quantum refinements of bacteriorhodopsin. The 2*mF*_o_ − *DF*_c_ maps are contoured at 1σ.

The deposited structure contains two conformations of retinal and Lys216, as can be seen in Fig. 1 ▸(*a*). The all-*trans* conformation of retinal is dominant (84% occupancy), with C15 (connected to Lys216) and C12 on opposite sides of the C13—C14 bond. The other conformation (16% occupancy) is 13-*cis* retinal with C15 and C12 on the same side. Trp182, which is located ∼2.8 Å from C20, also has two conformations with the same occupancies. HOH402, which receives a hydrogen bond from the Schiff-base N atom (2.7 Å N—O distance) is present only in the dominant conformation (but there is a positive difference density at the 3.1σ level next to it). In practice, the difference in the coordinates of the two conformations is quite small. The largest difference (throughout this section all comparisons are restricted to heavy atoms because the H atoms are mixed up in the deposited structure and it is sometimes hard to decide which H atoms correspond to each other in the two conformations) is 1.09 Å for C20, followed by 1.06 Å for C14, 0.75 Å for C15, 0.57 Å for C13 and 0.45 Å for NZ of Lys216 (the maximum-likelihood estimated coordinate error is 0.18 Å). The root-mean-squared deviation (r.m.s.d.) for all heavy atoms in the two residues is 0.38 Å (0.17 Å for Trp182). Therefore, it is quite hard to discern differences between the two conformations.

**Figure 2:**
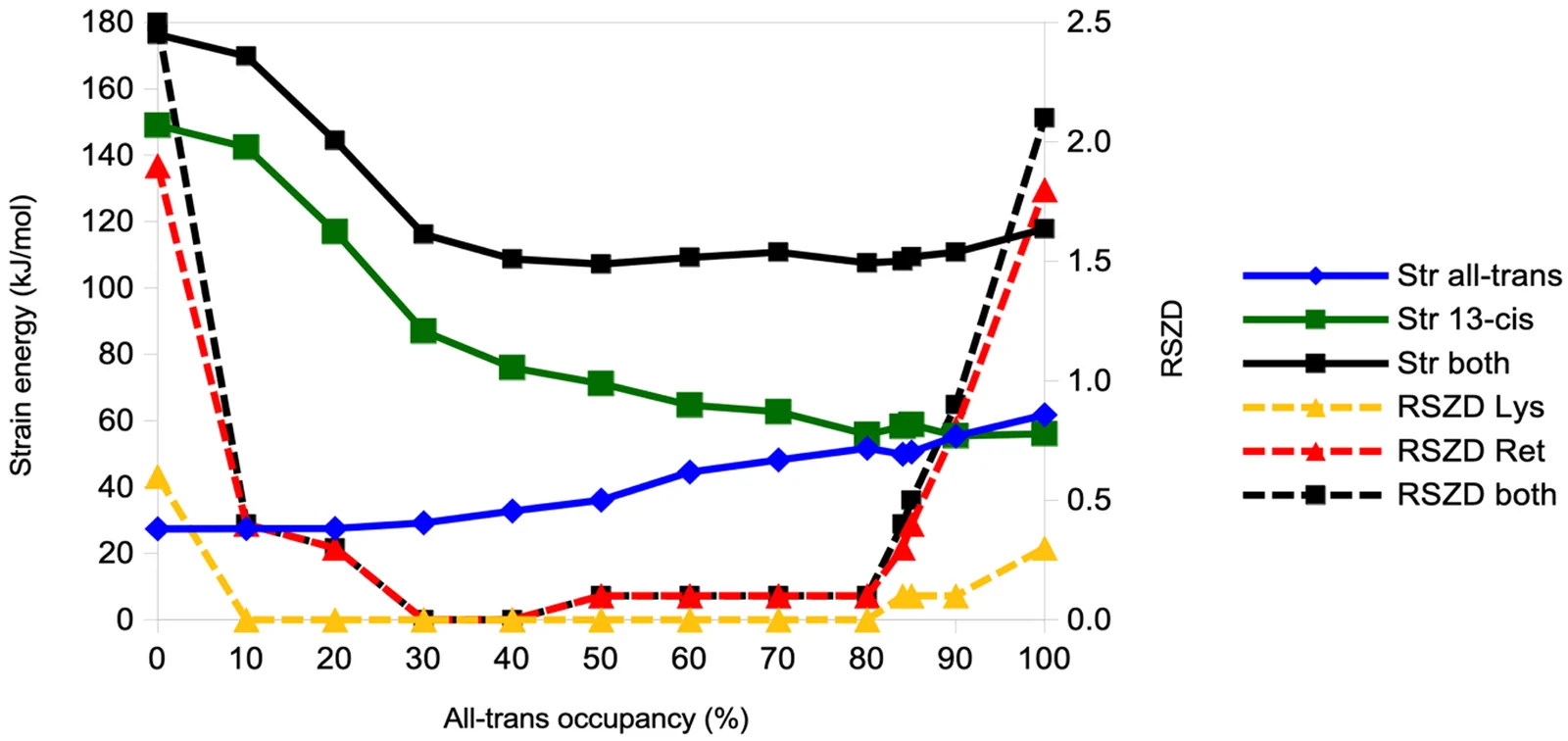
Strain energies (left axis; kJ mol^−1^) and RSZD scores (right axis) for QR of bacteriorhodopsin as a function of the occupancy of the all-*trans* conformation (the occupancy of the 13-*cis* conformation is always 100% minus this occupancy). Strain energies are shown for the all-*trans* conformation, the 13-*cis* conformation and for their sum. RSZD scores are shown for Lys216, retinal and their sum. Raw data are given in Supplementary Table S1.

We performed QR on the crystal structure with only retinal and Lys216 in the QM region (all atoms in retinal and atoms up to CD in Lys; see Fig. 1 ▸). To see how our quality measures depend on the occupation numbers, we ran a scan of occupation numbers of the two conformations in steps of 10%. It can be seen from Fig. 2 ▸ (raw data are given in Supplementary Table S1) that the RSZD scores of Lys216 and retinal are very low and essentially constant for occupancies of 30–80% for the all-*trans* conformation (and therefore 70–20% for the 13-*cis* conformation) at 0–0.1. However, at the end points the RSZD score of retinal is quite large at 1.8–1.9, although still below the threshold of 3 that would indicate an incorrectly modelled group.

The strain energy of the all-*trans* conformation increases steadily with its occupancy from 27 to 62 kJ mol^−1^, illustrating that the structure is a mixture of conformations (note that at zero occupancy the structure is determined entirely by the QM and restraints terms and not by the experimental data, *i.e.* a QM/MM calculation with the *Phenix* restraints as the MM function). Likewise, the strain energy of the 13-*cis* conformation also increases with its occupancy (and therefore decreases with the occupancy of the all-*trans* conformation). The strain energy of this conformation is higher and the variation is larger, from 54 to 149 kJ mol^−1^, reflecting that the structure is more distorted from the ideal vacuum structure for this conformation. As a result, the sum of the strain energies of the two QM regions is essentially constant for occupancies of 40–90% of the all-*trans* conformation, with a very shallow minimum at 50% occupancy.

This shows that the strain energy and RSZD scores are quite insensitive to the occupancies. Therefore, we decided to optimize the occupancies with a standard *Phenix* occupancy refinement (without any QM and without refining coordinates or ADPs) based on the QR structure. This gave an occupancy of 85% for the all-*trans* conformation and 15% for the 13-*cis* conformation, *i.e.* very close to the occupancies in the deposited structure. Finally, we repeated the QR calculation with these occupancies, leading to the structure shown in Fig. 1 ▸(*b*).

It can be seen that this QR structure is quite similar to the deposited structure (r.m.s.d. of 0.14 Å for the all-*trans* conformation and 0.13 Å for the *cis* conformation). Within the QR structure, the largest distance between the atoms of the two conformations of retinal is 1.08 Å for C20, followed by 0.98 Å for C14, 0.62 Å for NZ in Lys216, 0.53 Å for C13 and 0.49 Å for C10. The r.m.s.d. for the heavy atoms of the two conformations is 0.41 Å. The two C15—NZ distances are 1.34–1.35 Å, slightly longer than 1.31 Å in the deposited structure. On the other hand, the two NZ—CE distances in Lys216 are both 1.45 Å, slightly shorter than those in the deposited structure: 1.49–1.50 Å. In the QM-optimized structures (without any crystallographic data or protein surroundings), the C15—NZ and NZ—CE bond lengths are 1.34 and 1.46 Å for all-*trans* retinal and 1.35 and 1.46 Å for 13-*cis* retinal, respectively. Thus, these bonds are only minimally strained in the QR structures. The strain energies (for the full QM region) are much lower for the QR structure than for the deposited structure: 36 kJ mol^−1^ compared with 366 kJ mol^−1^ for all-*trans* and 71 kJ mol^−1^ compared with 473 kJ mol^−1^ for 13-*cis* retinal. The 13-*cis* conformation is 43 kJ mol^−1^ less stable than the all-*trans* conformation in the QR structure (115 kJ mol^−1^ in the deposited structure).

The RSZD score is 0.1 for Lys216 in the QR structure and 0.0 in the deposited structure, whereas for retinal it is 0.4 in the QR structure and 0.5 in the deposited structure. As shown in Fig. 2 ▸, the RSZD score is lower for both residues in QR structures, with more equal occupancies of the two conformations, and the occupancy optimized by *Phenix* is on the edge of where the RSZD score of retinal starts to increase.

If the structure is instead re-refined with *Phenix* using the same input as for QR (*i.e.* still with 85 and 15% occupancies), quite similar results are obtained. The RSZD score is 0.0 for both Lys216 and retinal. The strain energies are 1570 and 1674 kJ mol^−1^ before and 116 and 160 kJ mol^−1^ after optimizing the H atoms by QM for the all-*trans* and 13-*cis* conformations, respectively. The 13-*cis* conformation is 52 kJ mol^−1^ less stable than the all-*trans* conformation (113 kJ mol^−1^ without optimized H atoms). This shows that the current *Phenix* empirical restraints are closer to QM calculations than those used for the deposited structure, but the restraints for H atoms are poor (for a structure at the current resolution, the positions of the H atoms are determined entirely by the restraints). All structures indicate that 13-*cis* retinal is more distorted than all-*trans* retinal, but only QR gives an accurate estimate of the energy difference.

### 3.2. Xylose isomerase

Second, we studied xylose isomerase. This enzyme catalyses the reversible isomerization of an aldose to a ketose, for example xylose to xylulose or glucose to fructose. It uses two metal ions for substrate binding and catalysis, and a histidine residue (His54) for the catalytic hydride shift (Barreto *et al.*, 2025 ▸). In 2019, a time-resolved study of the binding and ring-opening of glucose was published for the enzyme from *Streptomyces rubiginosus* (Mehrabi, Schulz, Agthe *et al.*, 2019 ▸). The enzyme was studied at a temperature at which the reaction is very slow (20°C). The glucose substrate was delivered by shooting a liquid droplet onto a chip with protein crystals. Seven structures were obtained at times ranging from 0.015 to 60 s. The first structure already contained the substrate at 55% occupancy and the subsequent structures contained the substrate with occupancies of 60–65%, except that at 1 s, which had full occupancy. After 60 s, a ring-opened intermediate was observed at 62% occupancy. We have concentrated on the structure at 1 s (PDB entry 6qni), which best illustrates the capabilities of QR.

**Figure 3:**
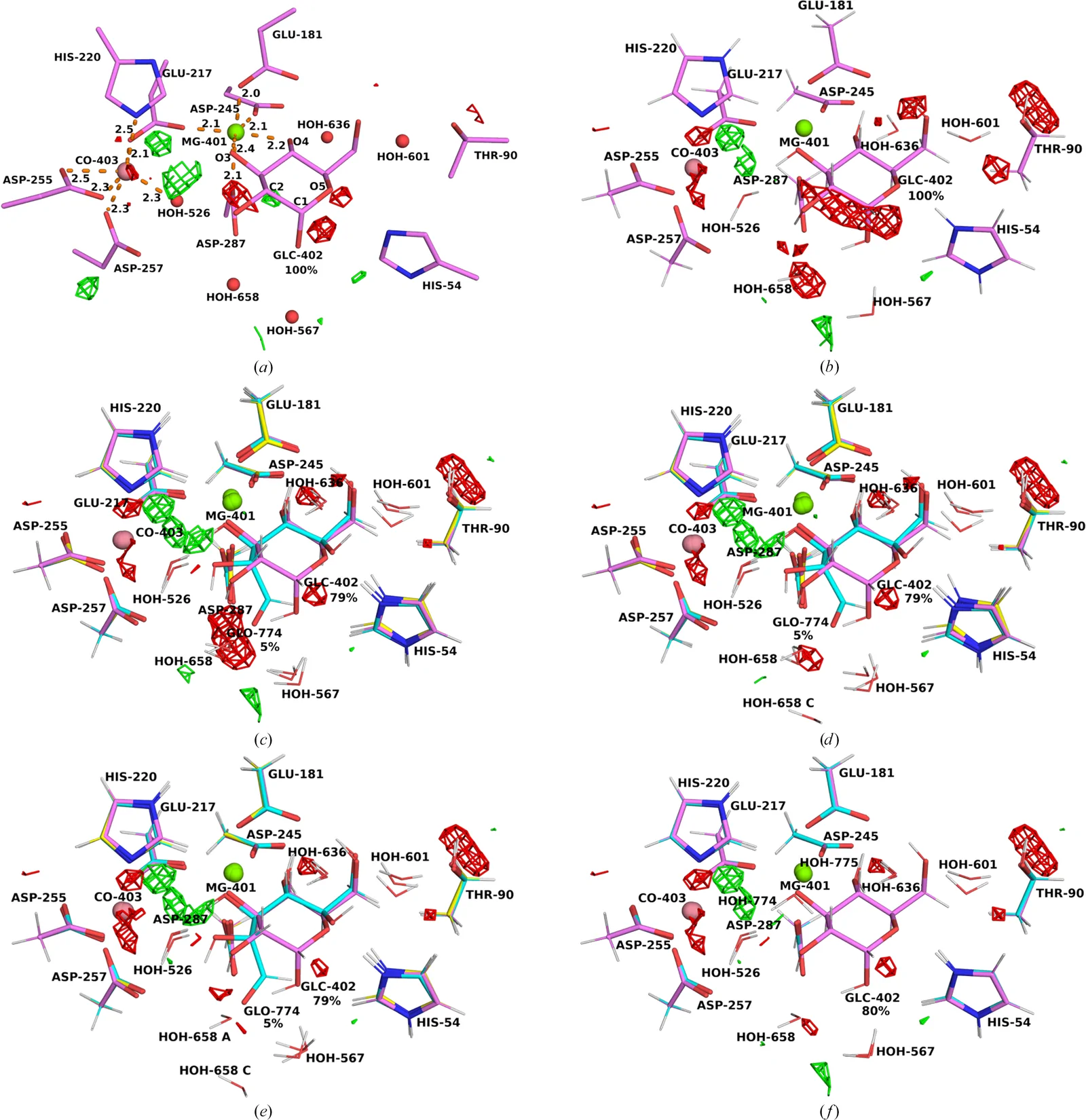
(*a*) Deposited structure of xylose isomerase (PDB entry 6qni). (*b*) QR structure with 100% Glc. (*c*) QR structure with 79% Glc, 5% Glo and 16% no substrate. (*d*) QR structure after movement of HOH658 in the 16% conformation. (*e*) QR structure after deleting HOH658 in the 5% conformation. (*f*) QR structure with 80% Glc and 20% no substrate. (*b*)–(*f*) illustrate the QM region used in QR. The conformation with Glc is depicted in pink, that with Glo in blue and that without substrate in yellow. The *mF*_o_ − *DF*_c_ difference maps are contoured at +3σ (green) and −3σ (red).

In the deposited structure [Fig. 3 ▸*(a*)], the two metal ions (one Mg and one Co) have full occupancy, as does the ring-closed substrate (Glc). The O3 and O4 atoms of the substrate coordinate to Mg with Mg—O distances of 2.39 and 2.24 Å, respectively. The Mg ion is also coordinated by four carboxy­late groups from Glu181, Glu217, Asp245 and Asp287, with Mg—O distances of 2.05–2.12 Å. The geometry is close to octahedral (the O—Mg—O angles are 75–108° or 160–165°), as is expected for Mg. The Co ion is also six-coordinate with four carboxylate atoms from Glu217, Asp255 (bidentate) and Asp257 (Co—O distances of 2.11–2.49 Å), one histidine ligand (His220, Co—N = 2.47 Å) and a water molecule (HOH526, Co—O = 2.27 Å). The structure is less regular (the angles around Co are 55–103° or 149–166°), mainly owing to the bidentate binding of Asp255. The structure shows significant positive difference densities around the Co ion and the HOH526 ligand [green meshes in Fig. 3 ▸(*a*); RSZD = 4.3 for both residues] and negative densities around Glc, especially near the O5, C1 and C2 atoms (red meshes; RSZD = 4.4, see Supplementary Table S2).

We performed QR on this structure, using a QM region that included the Mg and Co ions with all first-sphere ligands, as well as His54, Thr90 and four extra water molecules, HOH567, HOH601, HOH636 and HOH658 [see Fig. 3 ▸(*b*)]. All Asp and Glu residues were deprotonated; His220 was protonated on the N atom not binding to Co, whereas for His54 we tested two protonation states, with a proton on either both N atoms or only on NE2, which forms a hydrogen bond to O5 of glucose with a 2.7 Å N—O distance in the deposited structure. The two protonation states give similar results and only those with double protonation are discussed in the main text, whereas those with the singly protonated His54 are presented in Supplementary Tables S2 and S3 and Supplementary Fig. S2.

The quantum refinement gives results of a similar quality around the Co ion and HOH526 (RSZD = 4.8 and 2.6; Supplementary Table S2), whereas the negative densities around Glc are more concentrated at the C1—C2 bond (RSZD = 7.6). There is also a strong negative difference density around HOH658 [RSZD = 5.5; Fig. 3 ▸(*b*)]. HOH658 receives hydrogen bonds from the HO1 and HO2 atoms of Glc and from HOH567 and donates a hydrogen bond to HOH579. The Mg—O distances are 1.99–2.07 Å for the carboxylate atoms and 2.31 and 2.16 Å for O3 and O4 of Glc (Supplementary Table S3). For the Co ion, the distances are all ∼0.1 Å shorter than in the deposited structure, but still with a quite large variation: 2.04–2.21 Å for the carboxylate atoms (2.36 Å for the second carboxylate atom of Asp255), 2.21 Å for water and 2.38 Å for His220.

The large negative difference density around the C1—C2 bond of Glc indicates that the structure may actually also involve the ring-opened form. After some testing, we found that improved results could be obtained by modelling the site as a mixture of ring-closed Glc (79% occupancy), ring-opened glucose (Glo; 5% occupancy) and 16% without any substrate. The occupancies were obtained by optimizing occupancies in a normal (non-QR) refinement with *Phenix*, based on the QR structure (the coordinates and *B* factors were kept fixed), employing the complete QM region as an occupancy group (*i.e.* three occupancy groups with the same atoms, but with different coordinates and *B* factors, and differing in the treatment of the substrate). Then, we performed normal QR with fixed occupancies. Very little difference density is seen around the substrate [RSZD scores of 2.0 and 2.5 for Glc and Glo, respectively; Fig. 3 ▸(*c*)]. However, quite large positive difference densities are seen around HOH658 (RSZD = 4.2). Some improvement can be obtained by moving HOH658 into a nearby negative density for the 16% occupancy conformation [RSZD = 3.7, Fig. 3 ▸(*d*)]. A slight further improvement is seen in the maps if HOH658 is deleted from the 5% conformation [Fig. 3 ▸(*e*)]. This study illustrates how QR provided more easily interpreted electron density for the deposited structure (difference densities concentrated along the C1—C2 bond), leading to an alternative description of the structure involving both ring-closed and ring-opened conformations of the substrate. QR also provides improved restraints for the two metal ions.

Since 5% occupancy is very low and it is questionable whether the accuracy of the structure supports refinement of three conformations of the active site, we also tested a structure with only two conformations: 80% Glc and 20% empty site. This gave a structure with only slightly higher RSZD scores (1.5, 3.4, 1.2 and 4.0 for Co, Glc, HOH526 and HOH658, respectively), shown in Fig. 3 ▸(*f*). This structure is in better agreement with the other structures in the time series, which all showed partial occupancy of an empty active site.

To evaluate how much of the improvement comes from the actual QM calculations, we performed in parallel a similar investigation based only on re-refinement with the current *Phenix* software without QM, but otherwise employing the same input structures. The results are presented in Supplementary Fig. S3 and Supplementary Tables S2 and S3. Re-refining the deposited crystal structure also gave more concentrated negative difference densities, but they are not as concentrated on the C1—C2 bond as in QR, but also extend along the C1—O5 and C1—O1 bonds [Supplementary Fig. S3(*a*)], making the interpretation less clear. The coordinates differ between the QR and non-QM re-refinement by up to 1.4 Å for the H atoms of Glc and 0.37 Å for C1 of Glc.

The structure corresponding to that in Fig. 3 ▸(*c*) with three different conformations (79% ring-closed Glc, 5% ring-opened Glo and 16% empty site) is also rather similar, strongly reducing the difference densities around the substrate (Supplementary Fig. S3*b*). However, the SCR structure has additional negative difference density around the Co ion but less difference density around HOH658. The two structures with moved positions or occupancies of HOH658 give results of quite similar quality as QR (Supplementary Figs. S3*d* and S3*e*), although the positions of some atoms (especially those of the water molecules and the substrate) may differ by up to 0.9 Å for the H atoms and 0.7 Å for the heavy atoms. The same applies to the structure with only two conformations (Supplementary Fig. S3*e*).

### 3.3. Fluoroacetate dehalogenase

Fluoroacetate dehalogenase is an enzyme that catalyses the hydrolysis of C—F bonds with a turnover rate in the tens of seconds. It contains a catalytic triad consisting of Asp110, Asp134 and His280. In 2019, a time-resolved X-ray crystallo­graphic study of the enzyme was presented involving 18 structures at timepoints from (0 and) 30 ms to 27 s, indicating alternating half-of-the-sites reactivity and showing at least two reaction cycles (Mehrabi, Schulz, Dsouza *et al.*, 2019 ▸). The fluoroacetate (FAc) substrate binds to the active site in the *A* chain after 0.1 s, it reorients after 2.1 s, forms a covalent intermediate after 2.3 s and forms the product at 4.5 s, which is released at 6.2 s. Another substrate binds to the active site in the *B* chain after 2.3 s and forms an intermediate at 6.8 s. The product forms after 9 s and is released at 12 s. A new substrate is bound to the active site in chain *A* after 6.8 s and a second product is formed at 13.5 s.

**Figure 4:**
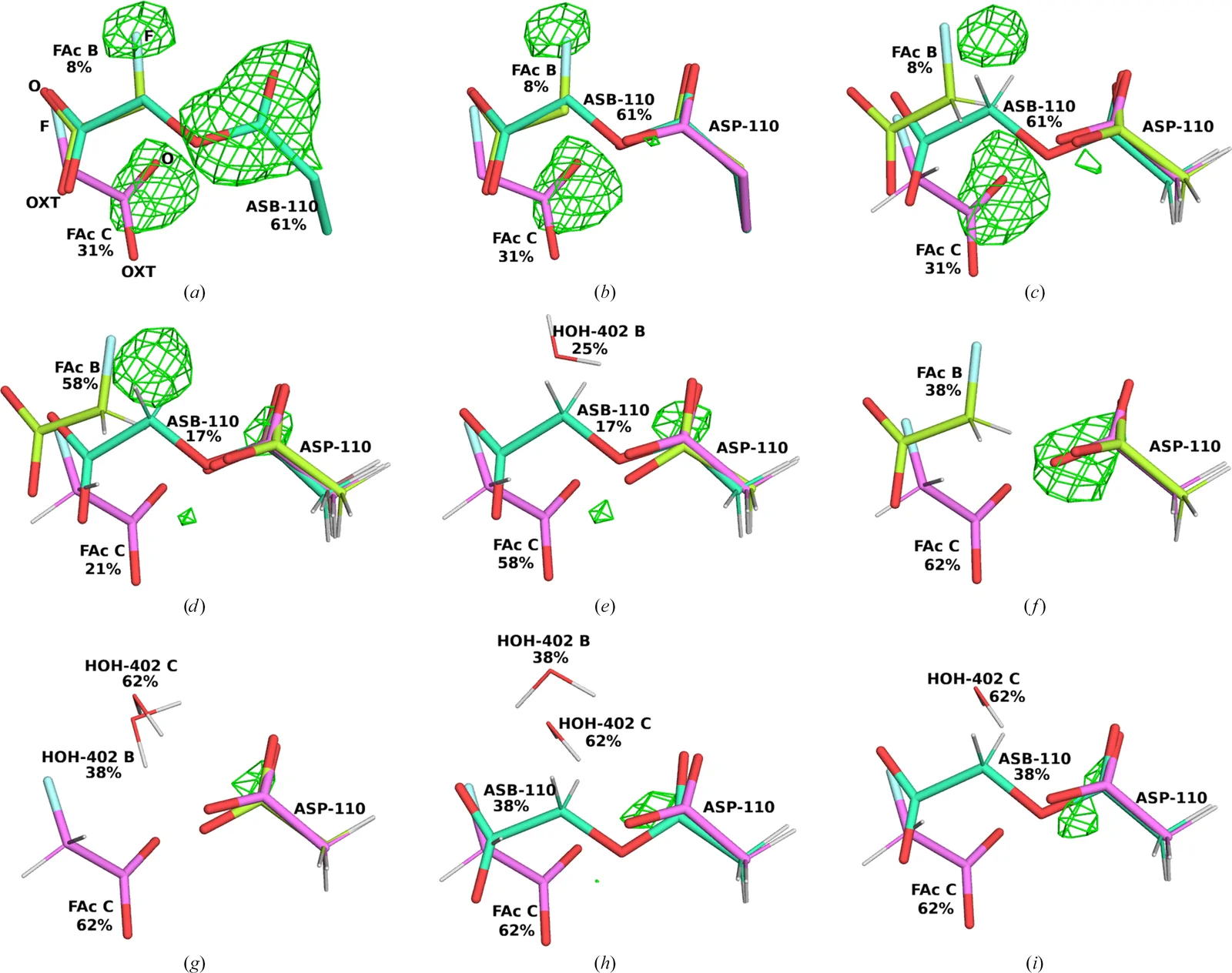
The deposited structure (PDB entry 6qhp) (*a*) and the various QR structures of the 2.3 s state of fluoroacetate dehalogenase. All structures show the active site of the *A* chain and are described in Table 1 ▸. (*c*)–(*i*) illustrate the QM region used in QR. The different conformations are colour-coded with the C atoms in dark green, light green, magenta and brown, respectively. The *mF*_o_ − *DF*_c_ difference maps are contoured at +3σ (green; no significant negative differences are seen in the structures).

We start by considering the most complicated active-site structure: the *A* chain in the structure at 2.3 s (PDB entry 6qhp; 1.8 Å resolution), which according to the deposited structure shows a mixture of the substrate in two different orientations (8% and 31% occupancy, respectively), as well as a covalent intermediate between Asp110 and the defluorinated substrate (61% occupancy; called Asb in the structure). As can be seen from Fig. 4 ▸(*a*), there is a significant overlap between the three conformations of the substrate atoms. In particular, all atoms of FAc in the B conformation overlap with the corresponding atoms in the covalent intermediate, except the F atom (which is not present in the covalent intermediate). Likewise, the fluoromethyl group of the C conformation of FAc overlaps with the carboxylate group of the other two conformations. The C conformation is essentially identical to the only substrate conformation in the structures obtained at short delays of 0.1–1.1 s. However, the structure at 2.1 s contains two new conformations of FAc, which differ mainly in the direction of the F atom. The O atom coincides with the same atom in the C conformation of the 2.3 s structure, whereas the OXT atom differs by 1.6–1.9 Å. The F atom in one of the two conformations is ∼0.8 Å from the OXT atom in the intermediate, whereas the F atom of the other conformation is 1.1 Å from the OXT atom in the C conformation of the 2.3 s structure. Thus, the density at the substrate binding site is quite crowded.

A peculiarity of the deposited 2.3 s structure is that Asp110 has only 61% occupancy (*i.e.* only the intermediate). This could be taken to indicate that the side chain of Asp110 has a different conformation or is mobile when the substrate is bound. However, the backbone also has only 61% occupancy, although the surrounding residues have full occupancy, which seems unreasonable. Moreover, structures at all timepoints have Asp110 in essentially the same conformation, even when the substrate is bound, so there is no support for any mobility of this side chain.

The deposited structure shows strong positive *mF*_o_ − *DF*_c_ difference densities around the carboxylate group of Asp110 (RSZD = 8.2; Supplementary Table S4), around the F atom in conformation B of FAc and around the carboxylate group of conformation C of FAc [RSZD = 4.8; shown in Fig. 4 ▸(*a*)]. The 2*mF*_o_ − *DF*_c_ density is weak for all conformations of FAc (also the intermediate), besides the carboxylate and F groups. QR of the deposited structure gives essentially the same results, with slight movements of FAc and with the same three prominent positive difference densities. The RSZD scores of Asb and FAc are 8.5 and 4.8, respectively.

Also including Asp110 in the two conformations with FAc (so that Asp110 has full occupancy) removes the positive difference density of Asp110 [Fig. 4 ▸(*b*); RSZD = 1.1], but does not improve the results around the two FAc conformations (RSZD = 4.9). The same applies to the corresponding QR structure [Fig. 4 ▸(*c*); RSZD = 1.4 and 4.6 for Asp110 and FAc, respectively]. Notably, FAc B moves away from Asp110 (by ∼1 Å) to decrease the repulsion between those two groups, illustrating a prominent difference between SCR and QR caused by the inclusion of electrostatics in the latter.

Next, we tested using four conformations with occupancies obtained by first running QR coordinate and (isotropic) ADP refinement, then *phenix.refine* (no QM) occupancy refinement and finally QR coordinate and ADP refinement again with the new occupancies. This gave 17% covalent intermediate (Asb110), 58% Asp110 + FAc conformation B, 21% Asp110 + FAc conformation C and 4% Asp110 without any FAc (Table 1 ▸; each conformation was treated as an occupancy group in the occupancy refinement and the four occupancies were constrained to sum to 100%). Again, FAc B moved away from Asp110 [Fig. 4 ▸(*d*)]. This led to a strong decrease in the positive difference density around the carboxylate group of FAc C, but an increase in the positive difference density near the F atom of FAc B, as a result of the movement of FAc B away from Asp110.

Therefore, we instead tried to model FAc B as a water molecule with the O atom close to the F atom of FAc B. We also deleted the fourth (minor) conformation with an empty active site. Thus, we employed three conformations: 17% Asb110, 25% Asp110 + water and 58% Asp110 + FAc C (Table 1 ▸). As can be seen in Fig. 4 ▸(*e*), this removed the positive density around FAc B (now HOH402). Only minor positive densities remained around the carboxylate groups of Asp110 and FAc C.

Next, we tried to further simplify the interpretation of the structure with only two conformations. First, we skipped the intermediate and included only Asp110 with the two different conformations of FAc: 38% B and 62% C. As with three conformations, this led to a movement of FAc B away from Asp110 [Fig. 4 ▸(*f*)]. This gave no prominent difference density on FAc, but a rather large positive difference density on the carboxylate group of Asp110. This is caused by a slight rotation of the carboxylate group of Asp110 to avoid repulsion with FAc B.

Therefore, we replaced FAc B with a water molecule and also added this water molecule in the FAc C conformation (still 38 and 62% occupancies). This gave the best results so far, with only a small positive density on the Asp110 carboxy­late group (smaller than in any of the structures discussed so far) [Fig. 4 ▸(*g*)].

Next, we tested the same structure, but with the intermediate (38% occupancy and still with the water molecule in both conformations). This gave a similar result [Fig. 4 ▸(*h*)], but with slightly more positive difference density on the Asp110 carboxylate group.

Finally, we included the water molecule together with FAc only (still 62% occupancy), not with the intermediate. This led to a slight movement of the intermediate towards the missing water molecule [Fig. 4 ▸(*i*)] and to the occurrence of two small positive densities around the carboxylate group of Asp110.

Thus, we can conclude that conformation B of FAc is better described by a water molecule and that there is no strong support for the intermediate. In fact, the best and simplest structure is obtained without any intermediate but with a fully occupied Asp110 and a water molecule, as well as a partly occupied FAc in conformation C [62% occupancy; Fig. 4 ▸(*g*)].

Again, we performed the same re-refinements without QM calculations in parallel. The results are shown in Supplementary Fig. S5. The structure with four conformations [corresponding to QR2 in Fig. 4 ▸(*d*)] shows a different position for FAc B, closer to Asp110 (owing to less repulsion; by 0.6 Å), giving less positive difference density (SCR2 in Supplementary Fig. S5). With FAc B replaced with a water molecule, the QR and SCR structures are more similar, but again the difference densities were smaller for the SCR structure, owing to a slight movement of the carboxylate group of Asp110 because electrostatics are missing in SCR [QR3 and SCR3 in Fig. 4 ▸(*e*) and Supplementary Fig. S5].

For the preferred QR structure [Fig. 4 ▸(*g*)], there are large differences in the position and orientation of the two water molecules replacing FAc between QR and SCR. In the latter, the two water molecules remain in the same location and orientation [SCR5 in Supplementary Fig. S5(*d*)], whereas in the QR structure they adapt electrostatically to the presence of FAc in one of the two conformations (movements of the H and O atoms of up to 1.3 and 0.6 Å, respectively). Likewise, the QR6 [Fig. 4 ▸(*h*)] and SCR6 (Supplementary Fig. S5) structures show extensive differences for the two conformations of HOH402, as do the QR7 and SCR7 structures for the single conformation of HOH402. This illustrates the importance of including electrostatics (as in QR) when placing important water molecules in enzyme active sites.

To show that QR can be used for the entire series of time-resolved structures, we applied QR to all 18 structures from the original study of fluoroacetate dehalogenase (Mehrabi, Schulz, Dsouza *et al.*, 2019 ▸). We concentrated on the active site and included only Asp110, the substrate/product/intermediate and two water molecules (one forming hydrogen bonds to Asp110 and Tyr219, called WatB in the following, and the other forming hydrogen bonds to the substrate and Tyr141, called WatC), if present in the deposited structures. Besides the starting resting structure, structures were considered only if they contain some FAc-derived molecule in the active site. On the other hand, we also considered the second subunit when it contained a substrate-derived ligand (structures collected 2.3–9 s after the trigger). All structures are described in Supplementary Table S5 and only the more interesting results are discussed here.

**Figure 5:**
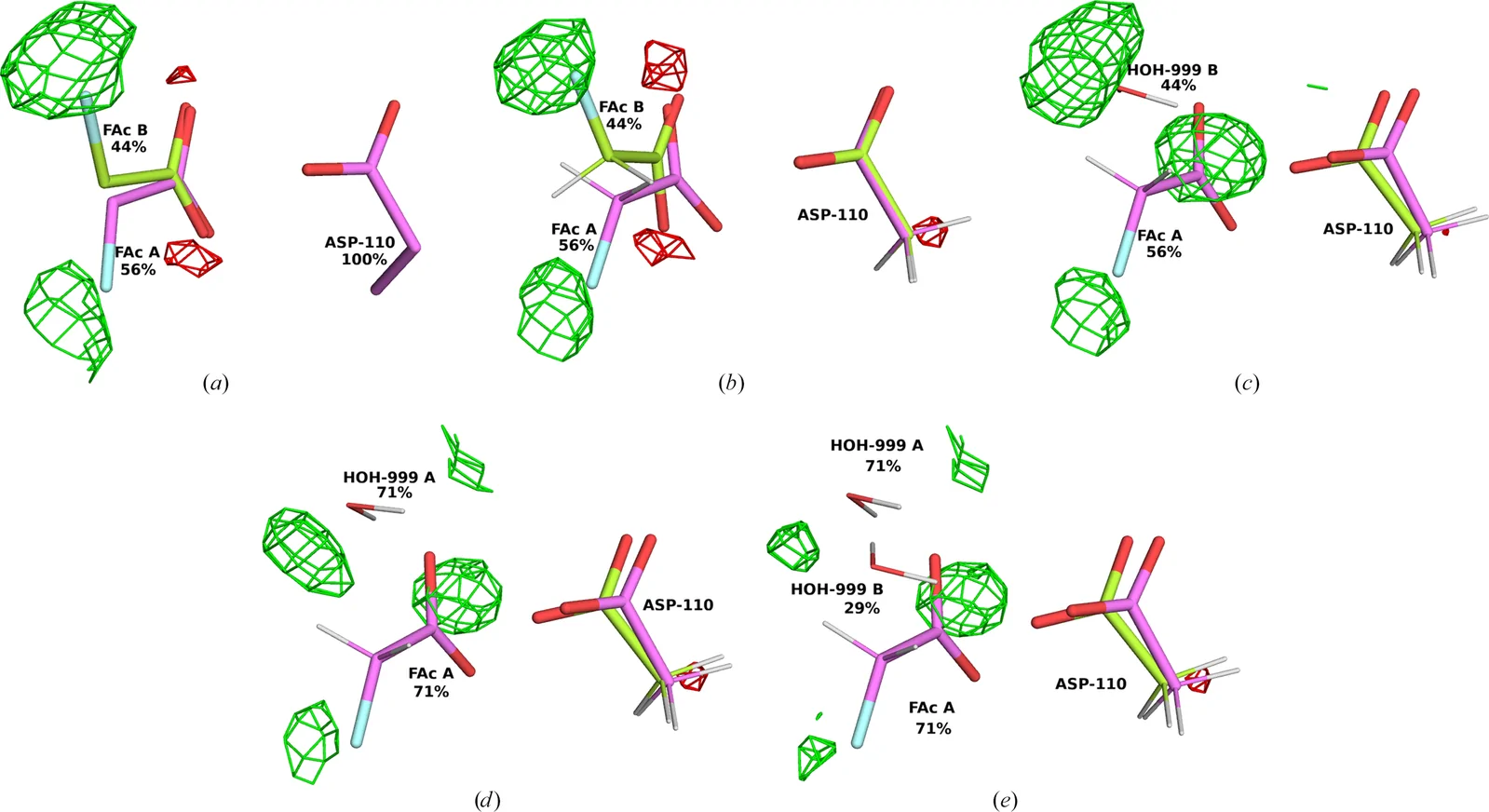
Various structures of the 2.1 s state of fluoroacetate dehalogenase (PDB entry 6gxt, chain *A*). (*a*) The deposited structure. (*b*) QR on the deposited structure (both with two conformations of FAc with 56% and 44% occupancy), (*c*) QR structure with 56% FAc and 44% HOH, (*d*) QR structure with 71% FAc + HOH and 29% empty site, (*e*) QR structure with 71% FAc + HOH and 29% HOH. The different conformations are colour-coded with the C atoms in green and magenta, respectively. (*b*)–(*e*) illustrate the QM region used in QR. The *mF*_o_ − *DF*_c_ difference maps are contoured at +3σ (green) and −3σ (red).

First, we considered the structures before and after PDB entry 6qhp in the time series. PDB entry 6gxt was collected 2.1 s after the trigger. The deposited structure was interpreted to contain FAc in two different conformations with 56% and 44% occupancy. However, as can be seen in Fig. 5 ▸(*a*), it contains significant positive difference density in the active site (RSZD of FAc = 5.8). The same applies to the corresponding QR structure [Fig. 5 ▸(*b*); RSZD = 4.8]. Replacing the minor FAc B conformation with water (as in our suggested interpretation of PDB entry 6qhp) makes the structure worse [Fig. 5 ▸(*c*); RSZD = 7.0]. However, the extensive positive difference densities indicate that FAc should have a higher occupancy. An (non-QM) occupancy refinement starting from the QR structure and moving the water molecule to the A conformation gives occupancies of 71% (FAc + HOH) and 29% (empty binding site). QR of this structure gives decreased difference densities [Fig. 5 ▸(*d*); RSZD = 4.1]. Finally, also adding the water molecule to the B conformation gives even better results [Fig. 5 ▸(*e*); RSZD = 3.2 for FAc and 2.5 for water]. The latter structure is our best interpretation of this structure and also consistent with our interpretation of PDB entry 6qhp. The corresponding SCR structures are shown in Supplementary Fig. S6, showing less variation in the position of Asp110 in particular (owing to the lack of electrostatics).

**Figure 6:**
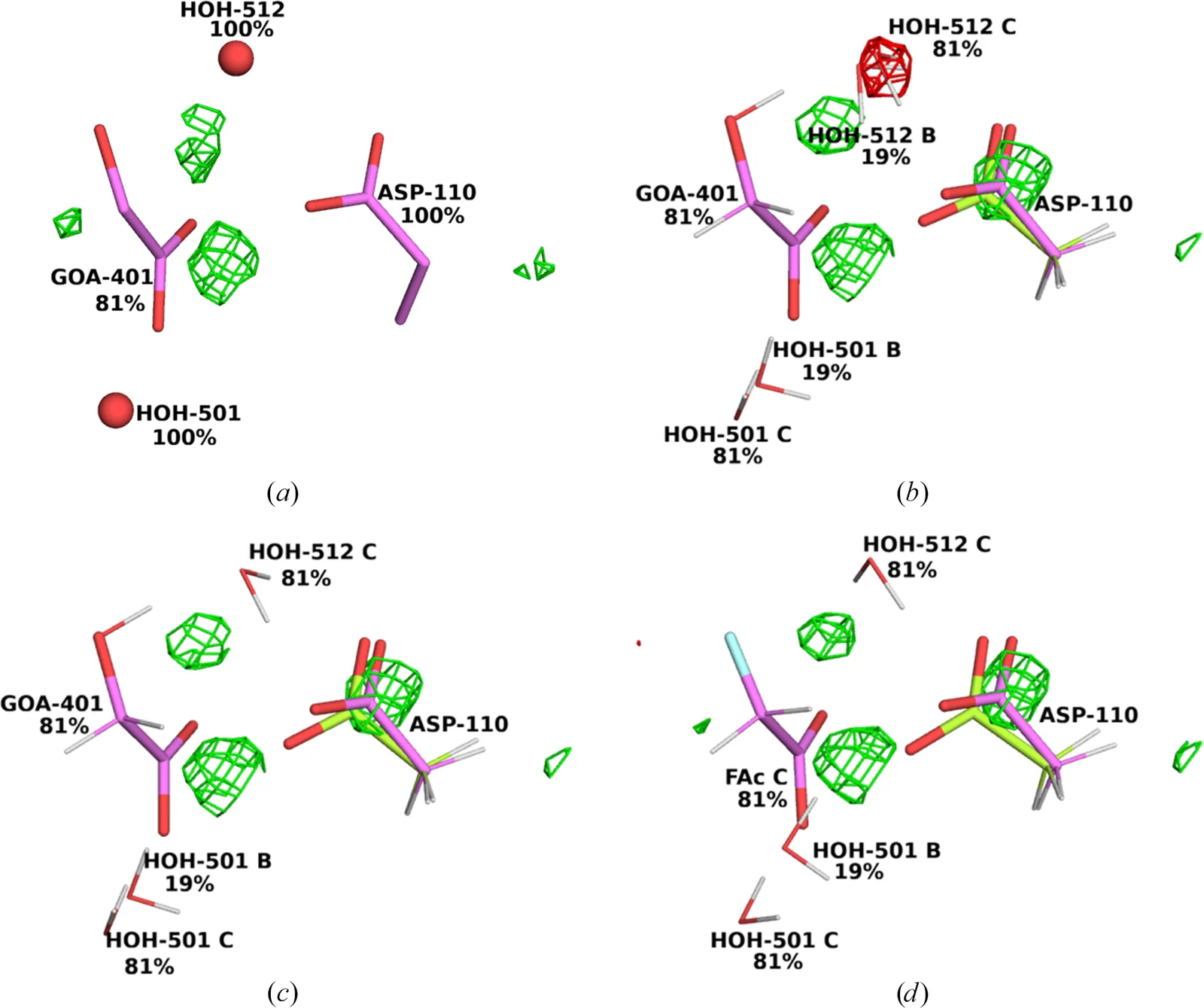
Various structures of the 4.5 s state of fluoroacetate dehalogenase (PDB entry 6qhw chain *A*). (*a*) The deposited structure, (*b*) QR on the deposited structure (both with 81% GOA and 19% empty site, and both conformations with two water molecules in the QM regions), (*c*) QR with only one water molecule in the B conformation, (*d*) QR with FAc instead of GOA. The different conformations are colour-coded with the C atoms in green and magenta, respectively. (*b*)–(*d*) illustrate the QM region used in QR. The *mF*_o_ − *DF*_c_ difference maps are contoured at +3σ (green) and −3σ (red).

Next, we turned to the structure at 4.5 s (PDB entry 6qhw). In the original study [Fig. 6 ▸(*a*)] the structure was interpreted as 81% glycolate product (GOA; CH_2_OHCOO^–^) and 19% empty binding site. This gives a rather good interpretation of the structure, with RSZD scores of 3.3 for GOA and 1.6–1.9 for two water molecules in the binding site (HOH501 and HOH512, both with full occupancy). In our first QR structure we employed the same occupancies and included both water molecules in the two conformations, as in the deposited structure. This gave rise to rather large difference densities on HOH512 [Fig. 6 ▸(*b*); RSZD = 3.8], because it moves slightly to form hydrogen bonds to the OH group of the product and the carboxylate group of Asp110. This could be cured by excluding this water molecule in one of the conformations [Fig. 6 ▸(*c*); RSZD = 2.8].

However, it should be pointed out that standard crystallo­graphy at the current resolution (1.72 Å) cannot discern between the FAc substrate and the GOA product. The only difference is that an F atom is replaced by an OH group, which are isoelectronic (including the H atom that is not explicitly modelled). In QR they are different, since the protons are included within the QM region. The OH group can form strong hydrogen bonds, both as a donor and an acceptor, whereas fluorine is only a hydrogen-bond acceptor. Therefore, we also tested replacing GOA with FAc with the same occupancy and number of water molecules. As can be seen from Fig. 6 ▸(*d*), this gives a similar fit to the experimental data, with RSZD scores of 2.0 and 2.6 for the two water molecules. It can be seen that FAc gives larger differences between the two conformations than GOA. Thus, we can conclude that there is actually no strong support for the observation of the product state. It might just as well be the substrate or a mixture of both. The corresponding SCR structures are shown in Supplementary Fig. S7, again showing less variation in the position of Asp110 and the water molecules.

Thus, even if the original suggestion with the substrate at 2.1 s, the intermediate at 2.3 s and the product at 4.5 s may seem attractive, it does not have much support from the experimental data. A more likely (but also less exciting) interpretation is that all three structures contain a mixture of an empty binding site and the substrate or the product in unknown proportions. This indicates that the intermediate is so short-lived that it does not build up to any significant amount in the crystal. Whether the product is observed or not depends on how fast it dissociates from the protein in the crystal and how fast the substrate binds again.

Next, we considered all the other structures in the time series. The results are described in Supplementary Table S5 and QR structures for the full time series are shown in Supplementary Fig. S8 (chain *A*) and Supplementary Fig. S9 (chain *B*). In the resting structure (0 s; PDB entry 6gxh), neither active site contains any substrate molecule. Instead, there are five water molecules in the active site in both chains. Two of them correspond to WatB and WatC, whereas the others replace the substrate. They form a network of hydrogen bonds between each other and to Asp110, Arg111, Arg114, Tyr141, Trp156 and Tyr219. QR reproduces the deposited structure [Supplementary Fig. S9(*a*)] but also indicates possible hydrogen positions (although there are many different possible positions of the H atoms).

Eight of the structures (those after 0.2–1.1, 6.8 and 9.0 s) contain a fully occupied FAc molecule in the chain *A* active site. For such pure structures (no mixtures), QR gives essentially the same result as the deposited structure or SCR. We attempted to model a partly occupied FAc molecule (and a partly occupied empty binding site) in many of the structures, but this gave occupancies of over 95% for FAc.

Three of the deposited structures contain a partly occupied product molecule in chain *A* (14 and 27 s) or *B* (9.0 s), similar to PDB entry 6qhw discussed above. For these, we tested the addition or removal of a water molecule in one conformation or the replacement of GOA with FAc. The results were similar to those obtained for PDB entry 6qhw. In particular, it is not possible to discern between the GOA product and the FAc substrate (in all structures, the RSZD score improves slightly if GOA is replaced by FAc, but in several structures this is compensated by changes in RSZD for the other residues).

The deposited structure at 0.1 s (PDB entry 6qhy) shows 52% FAc and 48% empty binding site. The structure can be slightly improved by adding three water molecules to the empty site (like the starting structure).

The deposited structure at 12 s (PDB entry 6qi1) shows 13% empty site and two conformations of FAc with occupancies of 45% and 42%. QR seems to confirm this structure. Reoptimizing the occupancies does not change the result and removing one of the FAc conformations and replacing it with a water molecule gives appreciably worse results.

Two structures of the *B* chain (at 2.3 and 4.5 s, PDB entries 6qhp and 6qhw) show two conformations of FAc (but no empty site). Both structures can be slightly improved by replacing one of the two conformations of FAc with a water molecule, similar to what we suggested for PDB entry 6gxt in Fig. 5 ▸.

The deposited structure at 6 s (PDB entry 6qhx) shows 55% FAc and 45% empty site in the *B* chain, which is a little unexpected since the previous two structures showed 100% FAc in two conformations with nearly the same occupancy. However, QR confirms this interpretation and it is actually in better agreement with our interpretation of the 2.3 and 4.5 s structures, indicating that they also may show a mixture of a single conformation of FAc and an empty binding site.

**Figure 7:**
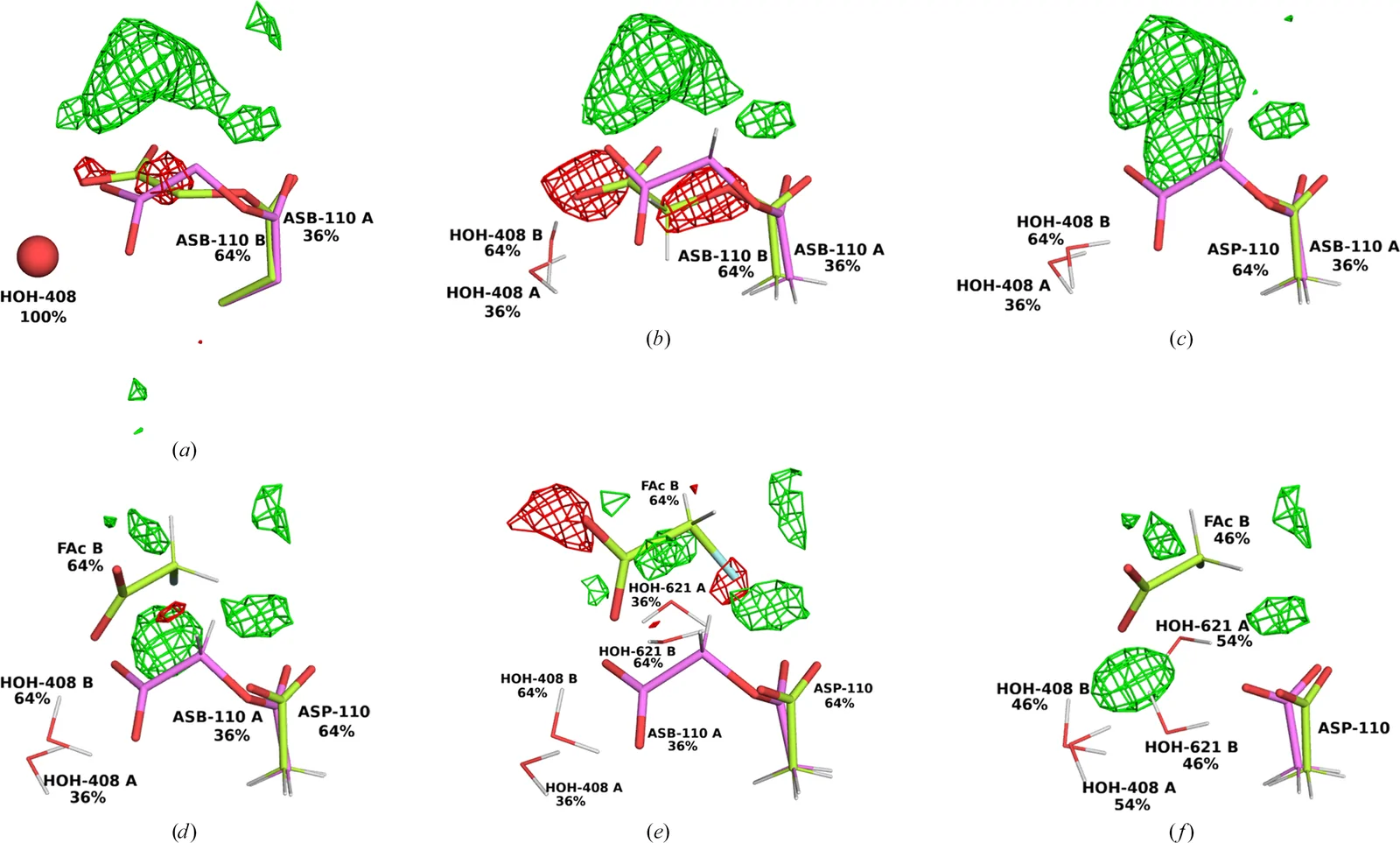
Various structures of the 7 s state of fluoroacetate dehalogenase (PDB entry 6qhz chain *B*). The structures are described in Table 2 ▸: (*a*) the deposited structure, (*b*) QR on the deposited structure (both with two conformations of the intermediate with 36% and 64% occupancy, and both conformations with one water molecule in the QM regions), (*c*) QR without the B conformation of the intermediate, (*d*) QR with FAc inserted for conformation B, (*e*) the same as (*d*) but with an extra water molecule included in both conformations, (*f*) the same as (*e*) but without the intermediate also in conformation A and with new occupancies of 54% and 46%. All structures show the active site of the *B* chain. The two conformations are colour-coded with the C atoms in light green and magenta, respectively. The *mF*_o_ − *DF*_c_ difference maps are contoured at +3σ (green) and −3σ (red).

The structure obtained after 7 s (PDB entry 6qhz) is the most interesting structure for the *B* chain. In the deposited structure it was interpreted as a 36/64% mixture of two different conformations of the Asb covalent intermediate [Fig. 7 ▸(*a*)]. In the 64% conformation both carboxylate atoms of the intermediate form hydrogen bonds to Arg114, one extremely short (2.17 Å) and the other more normal (2.72 Å). In the other conformation, one carboxylate atom also forms a hydrogen bond to Arg114 (2.34 Å; it is only 0.52 Å from the carboxylate atom of the other conformation), whereas the other carboxylate atom forms a hydrogen bond to HOH408 (which forms a hydrogen bond with Tyr141). There is no continuous electron density for the intermediate at the 1.5σ level. There are extensive positive and negative difference densities around the outer part of the intermediate, in particular negative densities around the very short hydrogen bond to Arg114 and around the C1—C2 bond of the intermediate in both conformations, as well as a large positive density between the intermediate and His155, Trp156 and Tyr219.

QR did not change the structure much, but moved the outer part of the two conformations of the intermediate somewhat further away from each other [Fig. 7 ▸(*b*)]. This did not improve the difference densities but moved one of the negative densities further towards the C2—OD1 bond of the 64% conformation of the intermediate.

Next, we removed the intermediate from the 64% conformation. This removed all the negative difference densities but increased the positive difference densities [Fig. 7 ▸(*c*)]. Therefore, we built FAc into the major positive density (64% occupancy). This removed most of the positive density, except the part that appeared when the second conformation of the intermediate was removed [Fig. 7 ▸(*d*)].

Consequently, we inserted a water molecule into this density in both conformations. This removed the positive density, but it induced a rotation of the FAc molecule that is not supported by the electron density [Fig. 7 ▸(*e*)]. Finally, we also removed the second intermediate, keeping FAc in one conformation together with two water molecules (46% occupancy), and with only the same two water molecules in the other conformation (54% occupancy). This led to slightly improved difference densities, although there are still some scattered positive difference densities [Fig. 7 ▸(*f*)]. It is probably possible to further improve the structure with additional partly occupied water molecules, but it is questionable whether the resolution of the structure is sufficient to support three different conformations of water molecules. The key point is that the structure can be interpreted at least as well without any intermediate, as in chain *A*.

As for all of the other structures, in parallel we also performed standard *Phenix* (non-QM) refinement with the same interpretations. The results in Supplementary Fig. S10 show that results of a similar quality are also obtained without QM. The positions of Asp110, Asb and FAc are similar, but the positions of water molecules and especially their H atoms differ considerably owing to the missing electrostatics in SCR. In the poorly defined electron density of the mixture structures in the time-resolved data, the hydrogen-bond structure of the water molecules is key to proper interpretation.

## 4. Conclusions

In this study, we have tested the potential of using QR to interpret time-resolved crystallographic data. QR is similar to standard crystallographic refinement, but the empirical restraints that are used to supplement the experimental data and ensure chemically reasonable structures are replaced by QM calculations, which provide a more accurate description of the site of interest that automatically adapts to the actual structure and provides detailed interactions (hydrogen bonds, electrostatics, dispersion and repulsion) with the closest surroundings (within the QM region). This can be expected to be especially important for time-resolved data, which typically contain mixtures of several chemical species or conformations in the active site, for which the experimental data are hard to interpret and the empirical potential therefore becomes more important.

We have applied QR to three different proteins from previous investigations. For a structure of bacteriorhodopsin, containing a mixture of all-*trans* and a distorted conformation of 13-*cis* retinal (Nango *et al.*, 2016 ▸), QR has a rather small influence on the structures but reduces the strain energies. For a structure of xylose isomerase (Mehrabi, Schulz, Agthe *et al.*, 2019 ▸), we provide an improved description of the observed densities, involving a partly empty substrate site and possibly both ring-closed and ring-opened conformations of the substrate. For fluoroacetate dehalogenase (Mehrabi, Schulz, Dsouza *et al.*, 2019 ▸), we study a complete time series of structures, showing that the QR results are consistent and give a new (albeit somewhat less exciting) interpretation of the structural events in the reaction. In particular, we point out that there is no strong support for the reaction intermediate and that it is often hard to discern between the substrate and the product of the reaction.

The three data sets we have worked on vary in terms of experiments, data quality and software used. In particular, the xylose isomerase data set has the highest multiplicity, whereas that of fluoroacetate dehalogenase has both the lowest multiplicity and overall *I*/σ(*I*) ratio. In principle, QR can be used for any crystallographic structures, but with lower data quality it becomes harder to discriminate between various structural interpretations. This probably explains why it was often hard to discern between various interpretations of the fluoro­acetate dehalogenase structures.

When interpreting mixtures, it is important to obtain occupancies for the various conformations. For bacterio­rhodopsin, we tested obtaining these by QR, scanning through 11 different values and deciding which is best by studying the RSZD scores and the strain energies [as was performed previously to decide the *w*_x_ weight factor in equations (1) and (2) (Lundgren *et al.*, 2024 ▸)]. However, the structure was insensitive to the occupancies (besides the largest and smallest values). Therefore, we instead allowed *phenix.refine* to optimize the occupancies starting from QR structures and then performed the final QR with fixed occupancies.

For all structures, we performed standard crystallographic (non-QM) refinement in parallel to check whether QM is really needed for the interpretations. In many cases SCR gives similar structures, for example for bacteriorhodopsin. However, the strain energies are significantly different and the interpretation of the QR structures is easier (difference densities are more concentrated on certain bonds). Moreover, SCR differs from QR in that electrostatic interactions are missing, leading to substantial differences in the refined coordinates of water molecules and other polar groups. In the interpretation of mixtures, it is essential to also properly model electrostatic interactions.

Altogether, this study illustrates that QR is a useful approach to study time-resolved data, especially when the electron density is ambiguous, for example when there is a mixture of several states. QR can then give improved structures, more reliable interpretations of the structures and much more accurate strain energies. QR (like standard refinement) should be performed for several conceivable interpretations of the structure to decide which model gives the best and simplest description of the experimental maps. Naturally, QR (again like standard refinement) still needs background knowledge to suggest which chemical species may be present in the structure, but once this is done QR provides an ideal description for mixtures of several states, for which the exact coordinates are almost entirely determined by the empirical restraints or the QM calculations.

Naturally, QR is computationally more demanding than standard crystallographic refinement and the time consumption strongly depends on the size of the QM region. The quantum refinements took ∼30 min on a single core for fluoro­acetate dehalogenase, ∼60 min on four cores for bacteriorhodopsin and ∼7 h on 32 cores for xylose isomerase (on an Intel Xeon Gold 6130 CPU). Finally, it should be pointed out that QR is not restricted to X-ray crystallographic data but works equally well for all radiation sources supported by the *Phenix* software, also including, for example, cryogenic electron microscopy, neutron crystallo­graphy and electron crystallography (Lundgren *et al.*, 2024 ▸, 2026 ▸).

## Supplementary Material

PDB files of the QM system for all QR structures. DOI: 10.1107/S2052252526009486/rs5010sup1.zip

Supplementary tables and figures. DOI: 10.1107/S2052252526009486/rs5010sup2.pdf

## Figures and Tables

**Table 1:** The various structures studied for the 2.3 s state of fluoroacetate dehalogenase (PDB entry 6qhp, chain *A*) The table describes conformations (Conf 1–4) employed in the active site. ‘Occ’ is the occupancy in % (they always sum to 100%), ‘Res’ is the residue(s); Asp or Asb is always residue 110. HOH is always water 402. ‘Dep’ is the deposited structure. Each conformation was treated as an occupancy group (with the same occupancy) in the occupancy refinement.

		Conf 1		Conf 2		Conf 3		Conf 4	
Case	Figure	Occ	Res	Occ	Res	Occ	Res	Occ	Res
Dep	4(*a*)	61	Asb	8	FAc B	31	FAc C		
SCR1†	4(*b*)	61	Asb	8	FAc B	31	FAc C		
QR1	4(*c*)	61	Asb	8	FAc B	31	FAc C		
QR2	4(*d*)	17	Asb	58	Asp + FAc B	21	Asp + FAc C	4	Asp
QR3	4(*e*)	17	Asb	25	Asp + HOH B	58	Asp + FAc C		
QR4	4(*f*)			38	Asp + FAc B	62	Asp + FAc C		
QR5	4(*g*)			38	Asp + HOH B	62	Asp + FAc C + HOH C		
QR6	4(*h*)			38	Asb + HOH B	62	Asp + FAc C + HOH C		
QR7	4(*i*)			38	Asb	62	Asp + FAc C + HOH C		

† A non-QM refinement of the deposited structure, but also including Asp110 in conformations 2 and 3.

**Table 2:** The various structures tested for the 7 s state of fluoroacetate dehalogenase (PDB entry 6qhz, chain *B*) The table describes the two conformations (Conf 1 and 2) employed in the active site. ‘Occ’ are the occupancies in % (they always sum to 100%). W is always HOH. ‘Dep’ is the deposited structure.

		Conf 1		Conf 2	
Case	Figure	Occ	Residues	Occ	Residues
Dep	7(*a*)	36	Asb + W408	64	Asb + W408
QR1	7(*b*)	36	Asb + W408	64	Asb + W408
QR2	7(*c*)	36	Asb + W408	64	Asp + W408
QR3	7(*d*)	36	Asb + W408	64	Asp + FAc B + W408
QR4	7(*e*)	36	Asb + W408 + W621	64	Asp + FAc B + W408 + W621
QR5	7(*f*)	54	Asp + W408 + W621	46	Asp + FAc B + W408 + W621
