# Supplementary material for: Quantum refinement can be used to improve time-resolved crystallographic structures
Source: IUCrJ. 2026 Nov 5;13(Pt 6):828–42. doi: 10.1107/S2052252526009486 (PMC13763905; doi:10.1107/S2052252526009486)

IUCrJ

Volume 13 (2026)

Supporting information for article:

Quantum refinement can be used to improve time-resolved crystallographic structures

Gayathri Yuvaraj, Kristoffer J. M. Lundgren, Esko Oksanen and Ulf Ryde

Table S1. Strain energies (kJ/mol), RSZD scores and *R* factors for the deposited, SCR and QR structures with different occupancies for bacteriorhodopsin (PDB ID: 5B6W). T = all-trans conformation, C = 13-cis conformation. Lys = Lys-216, Ret = retinal.

Structure	Occupancy		Strain		RSZD		R_{work}	R_{free}
	T	C	T	C	Lys	Ret		
Deposited	84	16	366	473	0.7	1.5	0.1609	0.1795
QR	0	100	27	149	0.6	1.9	0.1565	0.1790
QR	10	90	27	142	0.0	0.4	0.1566	0.1794
QR	20	80	27	117	0.0	0.3	0.1565	0.1793
QR	30	70	29	87	0.0	0.0	0.1574	0.1805
QR	40	60	33	76	0.0	0.0	0.1575	0.1805
QR	50	50	36	71	0.0	0.1	0.1561	0.1781
QR	60	40	45	65	0.0	0.1	0.1564	0.1786
QR	70	30	48	63	0.0	0.1	0.1561	0.1783
QR	80	20	57	54	0.0	0.1	0.1573	0.1804
QR	85	15	51	59	0.5	0.1	0.1569	0.1794
SCR	85	15	116[a]	160[a]	0.0	0.0	0.1454	0.1789
QR	90	10	55	55	0.1	0.8	0.1561	0.1776
QR	100	0	62	56	0.3	1.8	0.1562	0.1781

[a] The strain energies are 1570 and 1674 kJ/mol before QM optimisation of the positions of the H atoms, for the all-trans and 13-cis conformatios, respectively.

Table S2. Strain energies (kJ/mol) and RSZD scores for the deposited (dep), QR and SCR structures of xylose isomerase (PDB ID: 6QNI) with His-54 protonated either on only the NE2 atom (HIE) or on both ND1 and NE2 (HIP).

Str	Conformation			Strain			RSZD																		R_{work}	R_{free}
	A	B	C	A	B	C	H54	T90	E181	E217	H220	D245	D255	D257	D287	Mg	Co	Glc	Glo	W526	W567	W601	W636	W658		
dep	100% Glc			406			1.2	1.3	0.7	2.4	1.4	0.6	1.2	1.4	0.7	1.0	4.3	4.4		4.3	1.9	1.6	2.5	2.7	0.1664	0.2001
	**HIE-54**																									
QR1	100% Glc			239			1.2	2.5	0.5	2.5	1.7	0.2	1.2	0.6	0.2	0.6	4.6	7.7		2.6	2.3	2.5	2.3	5.3	0.1687	0.1989
QR2	79% Glc	5% Glo	16% No	242	157	272	0.7	1.0	0.3	1.3	1.4	0.0	0.9	0.2	0.0	0.1	2.2	1.6	2.7	1.1	1.8	1.2	1.8	3.7	0.1657	0.1996
QR3	79% Glc	5% Glo	16% No[a]	253	144	310	0.9	1.0	0.2	1.4	1.3	0.0	1.0	0.2	0.0	0.0	2.1	2.6	1.8	1.1	2.2	1.4	1.9	3.1	0.1654	0.1992
QR4	79% Glc	5% Glo[b]	16% No	234	118	243	0.8	1.0	0.2	1.2	1.2	0.0	1.0	0.2	0.0	0.1	2.9	2.3	1.8	1.0	1.8	1.2	1.5	3.8	0.1653	0.1993
QR5	80% Glc	20% No		412	180		1.0	1.2	0.3	1.2	1.5	0.1	1.1	0.3	0.1	0.2	2.6	1.5		1.2	1.0	1.2	1.8	4.2	0.1661	0.1991
	**HIP-54**																									
QR1	100% Glc			215			1.3	2.6	0.4	2.3	1.7	0.2	1.2	0.6	0.2	0.6	4.3	7.5		2.6	2.0	2.6	2.5	5.3	0.1687	0.1987
SCR1				475			1.4	2.6	0.4	1.8	1.7	0.9	1.2	0.6	0.3	0.5	4.3	7.2		2.3	2.7	2.5	1.9	4.5	0.1688	0.1988
QR2	79% Glc	5% Glo	16% No	219	139	277	0.7	1.1	0.1	1.4	1.4	0.0	0.9	0.2	0.0	0.1	2.2	2.5	2.0	1.1	1.2	1.1	1.5	4.2	0.1655	0.1993
SCR2				503	320	417	1.0	1.0	0.2	0.8	1.4	0.1	0.9	0.4	0.1	0.0	2.8	1.9	2.1	1.1	1.5	0.9	0.9	2.8	0.1653	0.1991
QR3	79% Glc	5% Glo	16% No[a]	219	128	277	0.7	1.1	0.1	1.4	1.3	0.0	1.0	0.2	0.0	0.1	2.3	2.6	1.3	1.3	1.4	1.2	1.5	3.7	0.1654	0.1994
SCR3				499	303	390	1.0	1.0	0.2	0.8	1.5	0.1	1.0	0.4	0.1	0.0	2.6	1.9	2.0	1.4	2.4	0.9	0.9	2.7	0.1653	0.1993
QR4	79% Glc	5% Glo[b]	16% No	218	104	258	0.8	1.1	0.2	1.4	1.3	0.0	0.9	0.2	0.0	0.1	2.9	2.6	1.9	1.1	1.5	1.2	1.3	3.5	0.1656	0.1996
SCR4				485	309	428	1.0	1.1	0.2	0.8	1.4	0.1	1.0	0.3	0.1	0.0	2.2	1.7	2.1	1.3	1.1	0.5	0.6	3.1	0.1655	0.1996
QR5	80% Glc	20% No		214	255		1.1	1.4	0.2	1.3	1.6	0.0	1.1	0.3	0.1	0.3	1.5	3.4	2.5	1.2	1.4	1.6	1.8	4.0	0.1660	0.1993
SCR5				482	404		1.2	1.3	0.3	1.0	1.6	0.1	1.1	0.4	0.2	0.1	2.7	2.3	2.3	1.4	1.3	1.1	1.1	2.8	0.1661	0.1990

[a] Another position of HOH-658 in conformation C.

[b] HOH-658 deleted from conformation B.

Table S3. Metal–ligand distances (Å) for the deposited, QR and SCR structures of xylose isomerase (PDB ID: 6QNI). O3 and O4 are atoms of glucose.

Structure	Conf	Mg						Co					
		E181	E217	D245	D287	O3	O4	E217	H220	D255	OD2	D257	W526
deposited	100% Glc	2.05	2.12	2.06	2.12	2.39	2.24	2.11	2.47	2.49	2.25	2.30	2.27
HIE-54													
QR1	100% Glc	2.05	2.00	2.00	2.07	2.29	2.14	2.04	2.36	2.39	2.17	2.19	2.21
QR2	79% Glc	2.05	2.02	1.99	2.08	2.28	2.12	2.03	2.37	2.40	2.18	2.20	2.21
	5% Glo	2.09	2.01	2.00	2.03	2.27	2.20	2.06	2.24	2.25	2.14	2.08	2.13
QR3	79% Glc	2.05	2.02	2.01	2.10	2.25	2.13	2.04	2.36	2.41	2.17	2.20	2.22
	5% Glo	2.10	2.01	2.01	2.02	2.27	2.19	2.06	2.23	2.25	2.14	2.08	2.13
QR4	79% Glc	2.09	2.00	2.02	2.03	2.24	2.16	2.07	2.19	2.25	2.14	2.10	2.12
	5% Glo	2.04	2.02	2.00	2.08	2.27	2.15	2.04	2.33	2.40	2.16	2.20	2.20
	16% No	2.07	2.06	2.05	2.04	2.29	2.17	2.05	2.21	2.27	2.14	2.11	2.13
QR5	80% Glc	2.07	2.03	2.05	2.07	2.27	2.12	2.04	2.28	2.29	2.13	2.13	2.16
	20% No	2.04	2.01	2.00	2.07	2.28	2.14	2.03	2.33	2.39	2.15	2.18	2.17
HIP-54													
QR1	100% Glc	2.04	1.99	2.01	2.07	2.27	2.14	2.04	1.99	2.01	2.07	2.27	2.14
SCR1	100% Glc	2.00	2.01	2.04	2.11	2.30	2.13	2.04	2.42	2.43	2.19	2.23	2.18
QR2	79% Glc	2.08	2.00	2.02	2.03	2.24	2.18	2.07	2.20	2.24	2.14	2.10	2.13
	5% Glo	2.05	2.01	2.01	2.08	2.24	2.14	2.04	2.35	2.41	2.17	2.21	2.21
SCR2	79% Glc	2.00	2.06	2.02	2.11	2.30	2.16	2.04	2.42	2.46	2.21	2.23	2.18
	5% Glo	1.99	1.95	2.27	2.05	2.26	2.19	2.02	2.40	2.39	2.12	2.18	2.20
QR3	79% Glc	2.07	2.01	2.02	2.03	2.26	2.23	2.06	2.22	2.26	2.13	2.11	2.15
	5% Glo	2.05	2.00	2.02	2.08	2.26	2.15	2.03	2.36	2.39	2.17	2.19	2.19
SCR3	79% Glc	2.00	1.95	2.25	2.05	2.25	2.17	2.03	2.37	2.39	2.14	2.18	2.18
	5% Glo	2.01	2.06	2.03	2.11	2.29	2.17	2.04	2.42	2.46	2.21	2.23	2.19
QR4	79% Glc	2.07	2.00	2.01	2.03	2.26	2.20	2.06	2.21	2.25	2.13	2.10	2.13
	5% Glo	2.04	2.35	2.61	2.41	2.16	2.20	2.18	2.35	2.41	2.16	2.20	2.18
	16% No	2.08	2.05	2.03	2.05	2.23	2.16	2.04	2.24	2.25	2.13	2.12	2.16
SCR4	79% Glc	1.97	2.01	2.25	2.05	2.35	2.26	2.02	2.43	2.51	2.09	2.23	2.21
	5% Glo	2.01	2.01	2.01	2.11	2.32	2.20	2.04	2.40	2.43	2.21	2.23	2.17
	16% No	1.97	2.00	2.20	2.07	2.35	2.14	2.02	2.43	2.49	2.11	2.22	2.21
QR5	80% Glc	2.07	2.04	2.03	2.07	2.24	2.14	2.05	2.26	2.26	2.13	2.13	2.15
	20% No	2.03	2.00	1.99	2.08	2.29	2.15	2.03	2.35	2.38	2.17	2.19	2.19
SCR5	80% Glc	1.96	1.99	2.19	2.08	2.34	2.07	2.02	2.44	2.49	2.12	2.22	2.21
	20% No	2.01	2.02	2.02	2.11	2.30	2.22	2.04	2.42	2.44	2.21	2.23	2.17

Table S4. Strain energies (kJ/mol), RSZD scores and *R* factors for the deposited structure (first two line) and the QR structures of fluoroacetate dehalogenase, time point 2256 ms (PDB id 6QHP). Asp = Asp-210, HOH = HOH-402.

Structure	Conformations				Strain				RSZD			R_{work}	R_{free}
	A	B	C	D	A	B	C	D	Asp	FAc	HOH		
Deposited	61% Asb	8% FAc	31% FAc		74	251	380		8.2	4.8		0.2186	0.2664
SCR2	61% Asb	8% FAc[a]	31% FAc[a]		41	193	537		1.1	4.9		0.2216	0.2661
QR0	61% Asb	8% FAc	31% FAc		39	2	1		8.5	4.8		0.2169	0.2652
QR1	61% Asb	8% FAc[a]	31% FAc[a]		34	142	113		1.4	4.6		0.2171	0.2657
QR2	17% Asb	21% Asp + FAc	58% Asp + FAc	4% Asp	23	112	155	1	3.2	3.1		0.2169	0.2669
SCR2					34	175	193	14	0.6	1.9		0.2168	0.2668
QR3	17% Asb	25% Asp + HOH	58% Asp + FAc		23	141	153		0.8	1.5	0.2	0.2170	0.2668
SCR3					35	161	190		0.8	1.2	0.3	0.2168	0.2668
QR4		38% Asp + FAc	62% Asp + FAc			143	159		2.6	1.1		0.2175	0.2668
SCR4						192	197		1.3	1.0		0.2176	0.2667
QR5		38% Asp + HOH	62% Asp + FAc + HOH			3	105		1.0	2.3	2.6	0.2176	0.2667
SCR5						20	167		0.6	2.2	0.8	0.2175	0.2666
QR6	38% Asb + HOH		62% Asp + FAc + HOH		39		107		1.7	1.4	2.1	0.2172	0.2667
SCR6					64		184		1.5	1.3	2.1	0.2172	0.2663
QR7	38% Asb		62% Asp + FAc + HOH		31		104		1.3	1.3	0.7	0.2173	0.2665
SCR7					37		145		0.9	1.1	0.6	0.2173	0.2664

[a] Asp-210 included also in conformations B and C

Table S5. Strain energies (str; kJ/mol), RSZD scores and *R* factors for the deposited (first line for each structure), the QR and SCR structures of the various structures of fluoroacetate dehalogenase. Asp = Asp-210, W = HOH. W997–W999 are new added water molecules.

Struct	Conf1	Conf2	Conf3	str1	str2	str3	str4	Asp	ASB	FAH	W1	W2	W3	W4	W5	R_{work}	R_{free}
Chain A																	
6gxh	100% 5Wat			197				1.5			1.3	2.0	4.3	1.6	2.3	0.1885	0.2373
QR1	100% 5Wat			132				1.4			2.9	2.9	2.8	1.3	2.0	0.1861	0.2366
SCR1				191				1.8			3.1	4.7	1.8	1.1	2.0	0.1861	0.2366
6qhy	52% FAc+W506+W524	48% W506+W524		274	96			1.6		3.2	2.1	4.3				0.1996	0.2327
QR1	52% FAc+W506+W524	48% W506+W524		182	81			1.5		3.3	2.4	2.2				0.1978	0.2332
SCR1				260	94			0.4		2.2	1.9	0.7				0.1976	0.2332
QR2	40% FAc+W506+W524	60% W506+W524+3Wat		159	87			1.1		3.7	3.1	2.4	2.0	3.1	3.2	0.1983	0.2336
SCR2				308	255			0.3		1.9	2.2	1.6	0.8	3.5	6.3	0.1980	0.2331
6qhv	100% FAc+W509+W513			225				1.1		1.5	1.8	0.7				0.1614	0.1941
QR1	100% FAc+W509+W513			133				1.6		1.7	3.6	3.0				0.1611	0.1940
SCR1				187				1.6		1.7	3.6	2.0				0.1610	0.1940
QR2	95% FAc+W509+W513	5% W509+W513+2Wat		107	78			1.1		2.0	2.9	2.9	0.2	0.2		0.1609	0.1941
SCR2				151	136			1.1		1.7	2.8	2.7	0.3	0.0		0.1607	0.1939
6qhu	100% FAc+W514+W515			204				4.3		2.0	0.9	0.9				0.1669	0.2068
QR1	100% FAc+W514+W515			134				5.2		2.5	2.3	2.1				0.1672	0.2069
SCR1				184				3.7		1.9	2.5	2.3				0.1671	0.2069
6qht	100% FAc+W502+W504			248				0.8		2.2	1.4	1.6				0.1687	0.2084
QR1	100% FAc+W502+W504			86				1.2		2.6	2.0	0.6				0.1683	0.2084
SCR1				230				0.9		2.5	2.4	4.2				0.1683	0.2085
QR2	100% FAc+W502+W504+W999			87				1.3		2.2	1.6	3.9	13.7			0.1682	0.2080
SCR2				579				0.9		1.7	2.5	3.9	10.7			0.1682	0.2083
QR3	87% FAc+W502+W504	13% W502+W504+W999		72	49			1.0		2.1	1.4	3.8	4.5			0.1684	0.2085
SCR3				262	125			0.8		1.4	1.7	4.4	4.2			0.1682	0.2087
6qhs	100% FAc+W501+W515			301				1.1		2.0	1.4	1.5				0.1604	0.1976
QR1	100% FAc+W501+W515			114				2.0		3.4	3.1	4.6				0.1620	0.1976
SCR1				366				0.9		2.6	3.3	4.0				0.1620	0.1975
6gxd	100% FAc+W554			159				0.5		2.1	1.2					0.1662	0.2109
QR1	100% FAc+W554			113				0.6		2.5	0.6					0.1653	0.2110
SCR1				140				0.6		2.0	0.8					0.1654	0.2111

QR2	100% FAc+W554+W999			119			0.6	2.5	0.3	5.0		0.1652	0.2106
SCR2				153			0.6	2.0	0.7	3.6		0.1651	0.2105
6qhq	100% FAc+W502+W524			184			0.5	1.6	2.6	1.4		0.1898	0.2294
QR1	100% FAc+W502+W524			131			0.7	3.0	5.6	3.4		0.1895	0.2293
SCR1				185			0.4	2.3	4.7	3.3		0.1893	0.2295
6gxt	56% FAc A	44% FAc B		214	250		0.8	5.4				0.1839	0.2293
QR1	56% FAc A	44% FAc B		174	170		1.0	4.8				0.1823	0.2277
SCR1				246	246		1.0	4.9				0.1823	0.2279
QR2	56% FAc A	44% HOH		59	49		0.8	6.9		7.0		0.1826	0.2284
SCR2				116	90		0.9	6.1		6.1		0.1825	0.2281
QR3	71% FAc+HOH	29% No		109	0		1.0	3.6			4.1	0.1821	0.2282
SCR3				208	15		0.8	2.5			2.3	0.1820	0.2280
QR4	71% FAc+HOH	29% HOH		105	29		1.0	3.2			2.5	0.1823	0.2280
SCR4				203	72		0.8	2.4			0.4	0.1821	0.2278
6qhw	81% GOA	19% No		263	96		1.5	3.3	0.8	1.8		0.1857	0.2273
QR1	81% GOA+2HOH	19% 2HOH		63	62		1.9	3.6	2.7	3.8		0.1867	0.2266
SCR1				285	85		1.0	3.5	1.5	3.9		0.1865	0.2267
QR2	81% GOA+2HOH	19% 1HOH		63	65		2.3	3.7	2.6	2.8		0.1867	0.2265
SCR2				299	91		0.9	3.6	1.5	2.4		0.1864	0.2268
QR3	81% Fac+2HOH	19% 1HOH		85	24		2.4	3.6	2.0	2.6		0.1866	0.2265
SCR3				338	88		0.9	3.6	1.6	2.4		0.1865	0.2268
6qhz	100% FAc+W511+W513			202			0.3	2.2	1.1	0.4		0.1845	0.2311
QR1	100% FAc+W511+W513			99			0.5	2.1	2.8	1.6		0.1843	0.2308
SCR1				161			0.5	1.9	1.7	1.7		0.1843	0.2307
6qi0	100% FAc+W508			151			1.3	2.5	0.9			0.1700	0.1998
QR1	100% FAc+W508			110			1.5	2.9	0.8			0.1703	0.1990
SCR1				150			1.1	2.5	1.4			0.1703	0.1996
QR2	100% FAc+W508+W999			131			1.5	2.7	0.8	4.2		0.1696	0.2001
SCR2				153			1.4	2.3	0.8	3.7		0.1696	0.2001
6qi1	45% FAc+W509	42% FAc+W509	13% W509	130	131	15	1.8	4.2		1.5		0.2121	0.2586
QR1	45% FAc+W509	42% FAc+W509	13% W509	65	75	3	1.6	3.6		1.8		0.2082	0.2592
SCR1				138	164	12	1.6	4.0		0.3		0.2079	0.2593
QR2	48% FAc+W509	44% FAc+W509	8% W509	67	54	3	1.6	3.7		1.8		0.2082	0.2592
SCR2				140	141	12	1.7	3.6		0.4		0.2078	0.2592

QR3	49% FAc+W509	51% W401+W509	16	77	1.7	6.0	6.4	1.5		0.2090	0.2592
SCR3			57	141	1.7	5.6	6.4	1.3		0.2089	0.2590
6qi2	64% GOA+W501	36% W501	212	98	0.8	2.4	1.7			0.1955	0.2355
QR1	64% GOA+W501	36% W501	149	52	0.7	3.3	1.8			0.1974	0.2352
SCR1			147	78	0.4	2.5	2.0			0.1969	0.2353
QR2	64% GOA+W501+W999	36% W501+W999	50	37	0.5	3.9	3.2	3.6		0.1971	0.2347
SCR2			97	71	0.2	2.8	2.0	4.9		0.1969	0.2353
QR3	64% FAc+W501	36% W501+W999	112	38	0.3	2.7	1.9	4.4		0.1970	0.2351
SCR3			140	69	0.2	3.0	2.0	4.1		0.1970	0.2354
6qi3	81% GOA+W512+W562	19% W512+W562	97	57	0.6	1.4	3.6	1.6		0.2019	0.2433
QR1	81% GOA+W512+W562	19% W512+W562	55	45	1.2	2.5	4.7	1.8		0.2027	0.2435
SCR1			123	57	0.1	1.6	4.9	1.2		0.2022	0.2438
QR2	81% GOA+W512+W562	19% W562	61	65	0.3	2.2	3.9	1.4		0.2025	0.2435
SCR2			104	71	0.3	1.8	3.8	1.8		0.2022	0.2438
QR3	81% FAc+W512+W562	19% W562	115	72	0.1	2.0	4.8	1.4		0.2025	0.2438
SCR3			142	72	0.2	1.8	3.8	1.7		0.2022	0.2436
Chain B											
6qhp	54% FAc+W525	46% FAc+W525	230	127	0.4	1.9	0.6			0.2185	0.2666
QR1	54% FAc+W525	46% FAc+W525	112	81	0.2	1.4	1.4			0.2172	0.2662
SCR1			197	157	0.2	1.9	0.1			0.2170	0.2662
QR2	54% FAc+W525	46% FAc+W401+W525	100	81	0.2	2.1	1.8			0.2181	0.2671
SCR2			175	172	0.2	1.6	0.4			0.2180	0.2670
QR3	61% FAc+W525	39% FAc+W401+W525	102	74	0.5	1.6	1.9			0.2181	0.2669
SCR3			174	175	0.2	1.4	0.4			0.2181	0.2670
QR4	61% FAc+W525	39% FAc+W401+W525	102	58	0.7	1.6	2.4			0.2182	0.2669
SCR4			170	162	0.2	1.3	0.4			0.2180	0.2669
QR5	61% FAc+W525+W999	39% FAc+W525+W999	144	81	0.5	1.3	1.1	0.7		0.2174	0.2669
SCR5			222	135	0.2	1.0	0.4	0.7		0.2173	0.2669
6qhw	50% FAc+W511+W543	50% FAc+W511+W543	207	132	1.0	0.8	3.2	1.6		0.1858	0.2276
QR1	50% FAc+W511+W543	50% FAc+W511+W543	148	94	0.8	0.9	4.2	1.6		0.1863	0.2271
SCR1			372	139	0.9	0.6	4.6	1.5		0.1862	0.2270
QR2	64% FAc+W511+W543+W704	36% W511+W543+W704	94	43	0.7	2.8	5.3	2.3	0.8	0.1867	0.2267
SCR2			345	95	0.7	2.8	3.7	1.8	1.3	0.1866	0.2266
QR3	59% FAc+W511+W543+W704	41% W401+W511+W543+W704	128	96	0.8	1.7	5.9	2.0	2.2	0.1876	0.2271

SCR3			378	152	0.8		1.3	4.2	0.4	1.6	0.1870	0.2269
QR4	59% FAc+W511+W543+W704	41% W401+W543+W704	92	57	0.7		1.9	3.7	1.8	0.7	0.1867	0.2265
SCR4			242	140	0.8		1.2	1.3	2.2	1.5	0.1865	0.2265
6qhx	55% FAc	45% empty	139	14	1.0		0.9				0.2235	0.2538
QR1	55% FAc	45% empty	82	1	0.0		0.5				0.2202	0.2537
SCR1			121	13	0.1		0.4				0.2200	0.2537
QR2	55% FAc+W999	45% W999	108	38	0.8		1.3	3.2			0.2190	0.2539
SCR2			155	49	0.1		0.4	1.7			0.2189	0.2539
6qhz	36% ASB+W408	64% ASB+W408	161	165		0.4		1.4			0.1846	0.2310
QR1	36% ASB+W408	64% ASB+W408	48	72		3.3		1.9			0.1846	0.2310
SCR1			76	105		4.1		0.8			0.1845	0.2311
QR2	36% ASB+W408	64% W408	44	86	3.2	0.7		1.2			0.1843	0.2312
SCR2			90	95	3.7	0.2		0.9			0.1841	0.2312
QR3	36% ASB+W408	64% FAc+W408	47	145	3.6	0.4	4.3	0.9			0.1841	0.2310
SCR3			95	209	4.1	0.2	3.8	0.7			0.1840	0.2311
QR4	36% ASB+W408+W999	64% FAc+W408+W999	58	466	1.0	0.9	5.4	0.6	0.6		0.1839	0.2312
SCR4			129	243	1.1	0.3	5.1	0.7	0.8		0.1838	0.2312
QR5	54% W408+W999	46% FAc+W408+W999	76	166	0.0		2.7	0.7	3.5		0.1844	0.2307
SCR5			103	487	0.4		2.4	0.8	2.2		0.1843	0.2308
6qi0	75% GOA	25% empty	178	18	0.6		2.8				0.1700	0.1998
QR1	75% GOA	25% empty	134	1	0.1		2.4				0.1699	0.1990
SCR1			207	13	0.6		1.6				0.1700	0.1992
QR2	75% FAc	25% empty	146	1	0.5		2.2				0.1700	0.1991
SCR2			195	14	0.6		1.7				0.1700	0.1991

Coordinates of the QM regions for all structures shown in Figures 1, 3–6 in PDB format are included in a separate file archive.

Figure S1. Convergence of R_{work} and R_{free} in a quantum refinement of the 2.3 s structure of fluoroacetate dehalogenase (PDB ID: 6QHP), the structure in Figure 4g (QR5), employing 10 macrocycles.

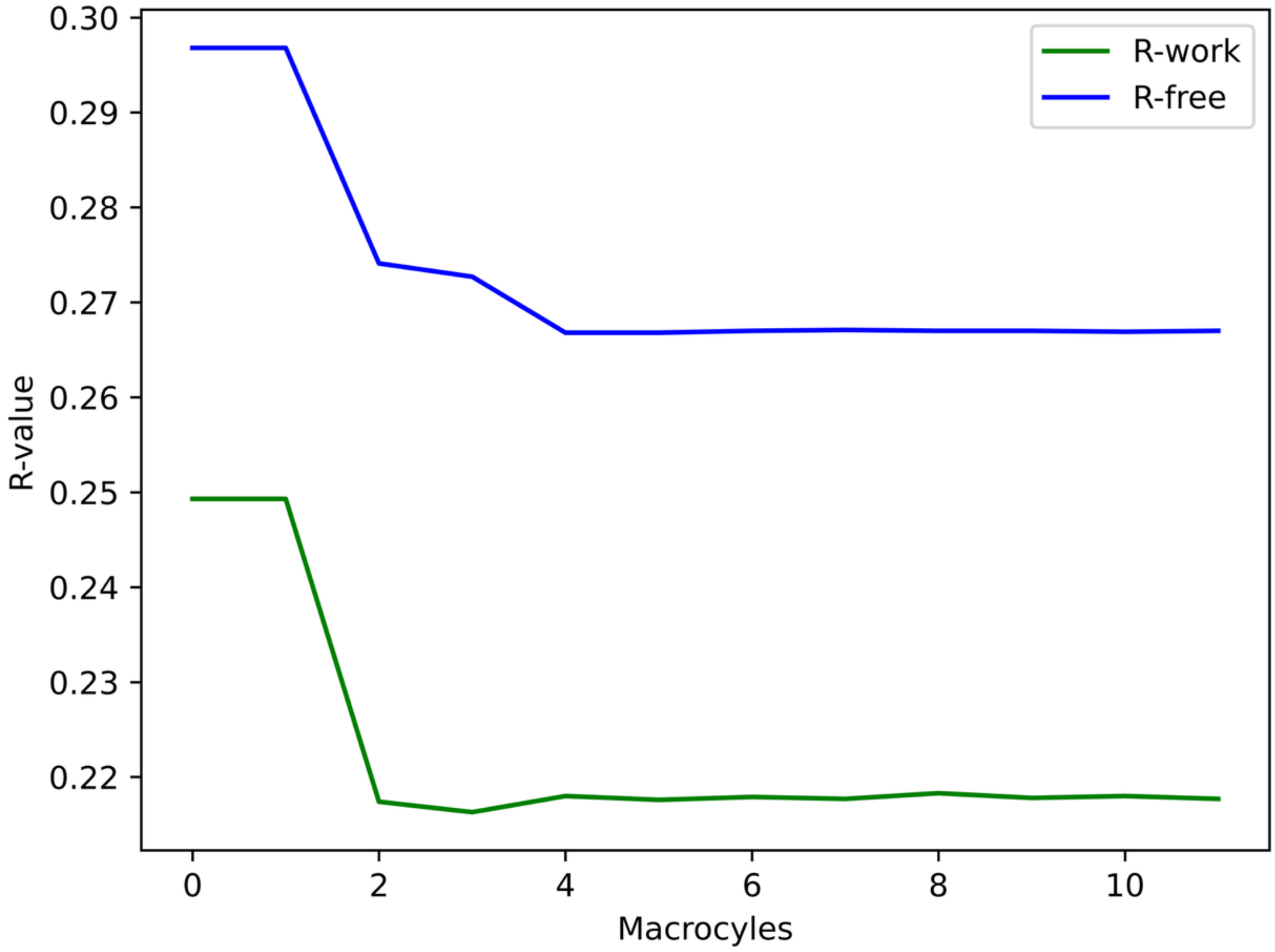

Figure S2. QR structures for xylose isomerase (PDB ID: 6QNI) with His-54 protonated only on NE2, showing the atoms in the QM regions. a) QR structure with 100% Glc. b) QR structure with 79% Glc, 5% Glo and 16% no substrate. c) QR structure after a movement of HOH-658 in the 16% conformation. d) QR structure after deleting HOH 658 in the 5% conformation. e) QR structure with 80% Glc and 20% no substrate. The different conformations are colour-coded on the C atoms in magenta, cyan and yellow, respectively. The $mF_o - DF_c$ difference maps are contoured at +3σ (green) and –3σ (red).

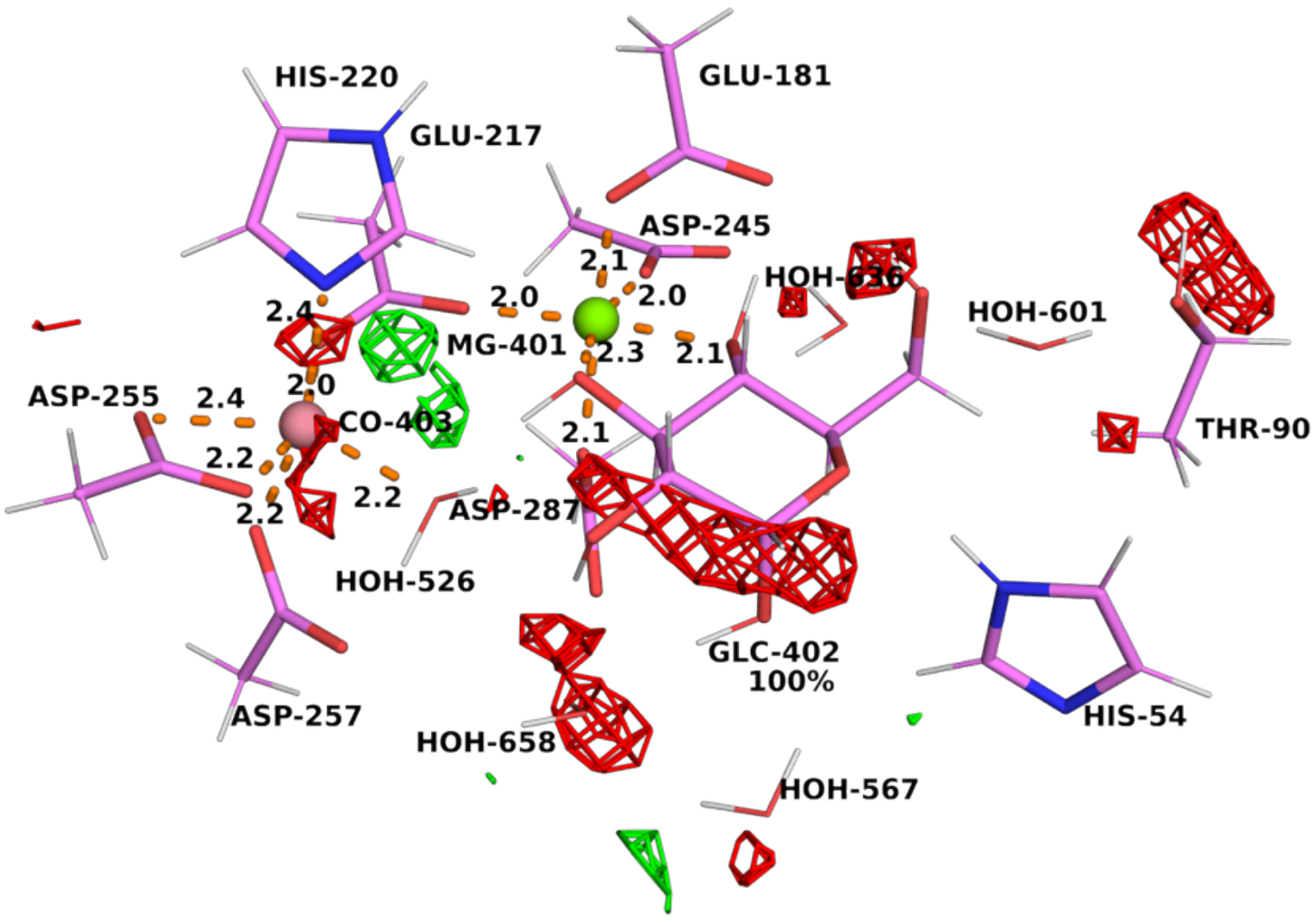

a

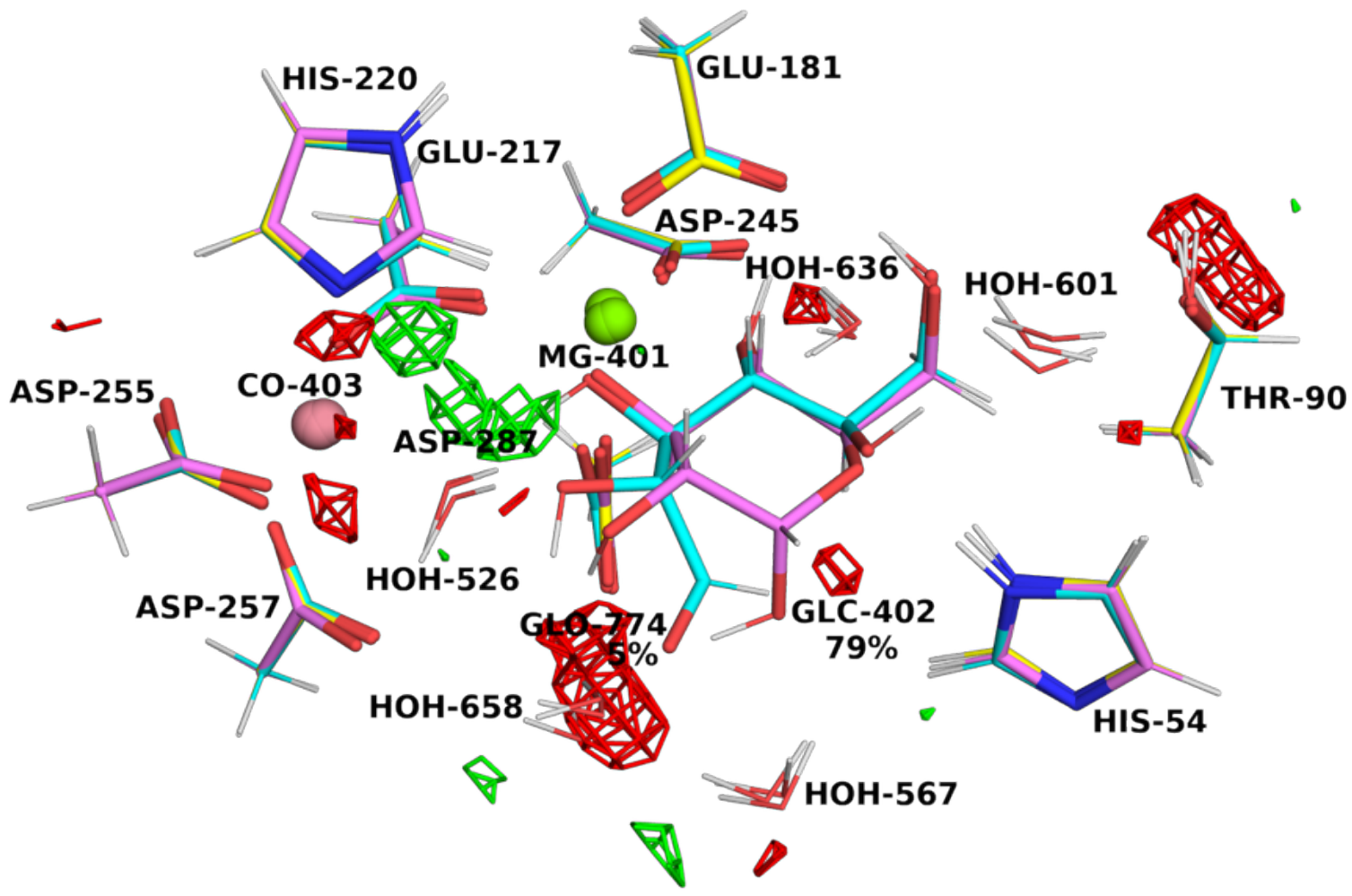

b

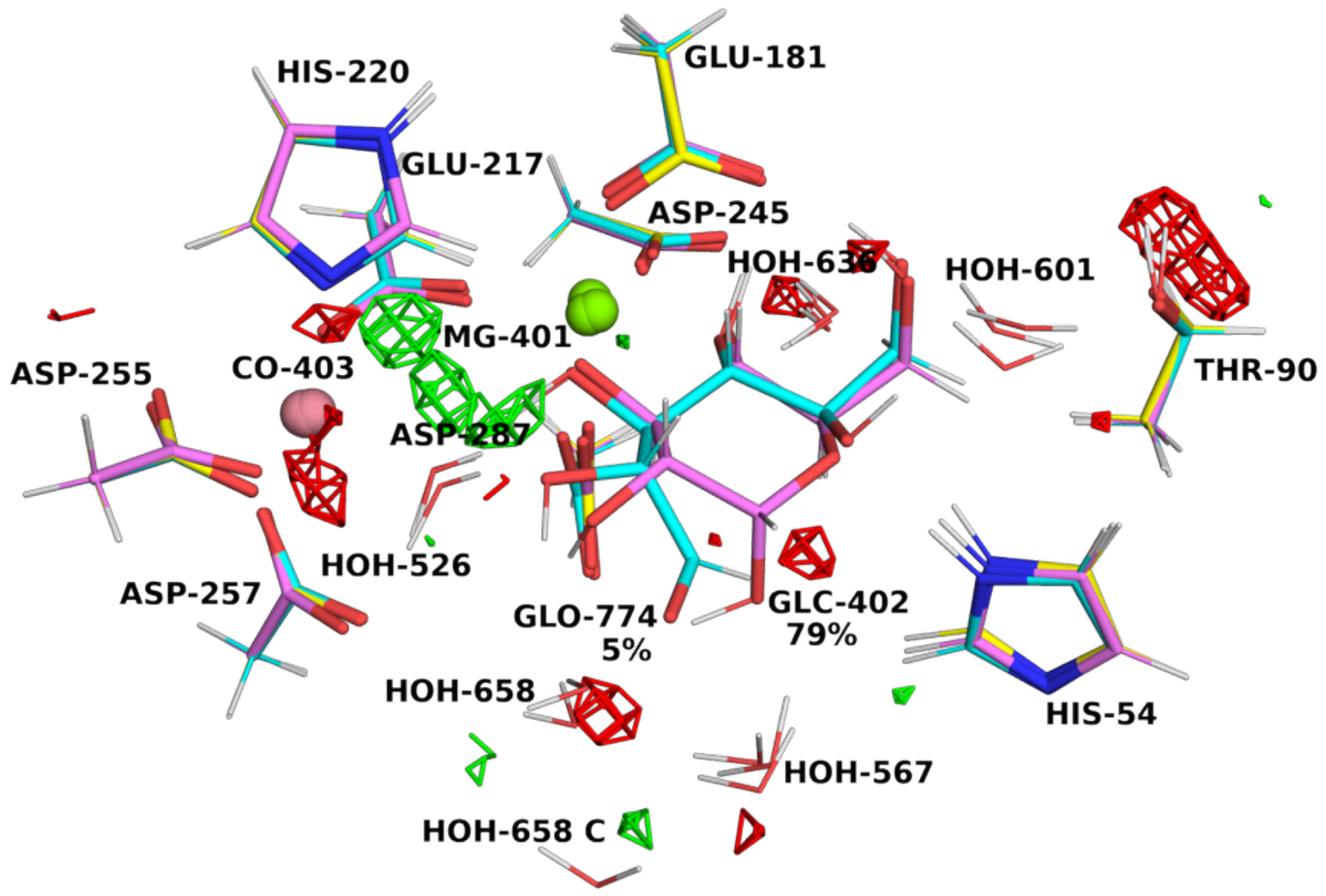
HIS-220
GLU-181
GLU-217
ASP-245
HOH-636
HOH-601
MG-401
CO-403
ASP-255
THR-90
ASP-287
HOH-526
ASP-257
GLO-774
5%
GLC-402
79%
HIS-54
HOH-658
HOH-567
HOH-658 C

c

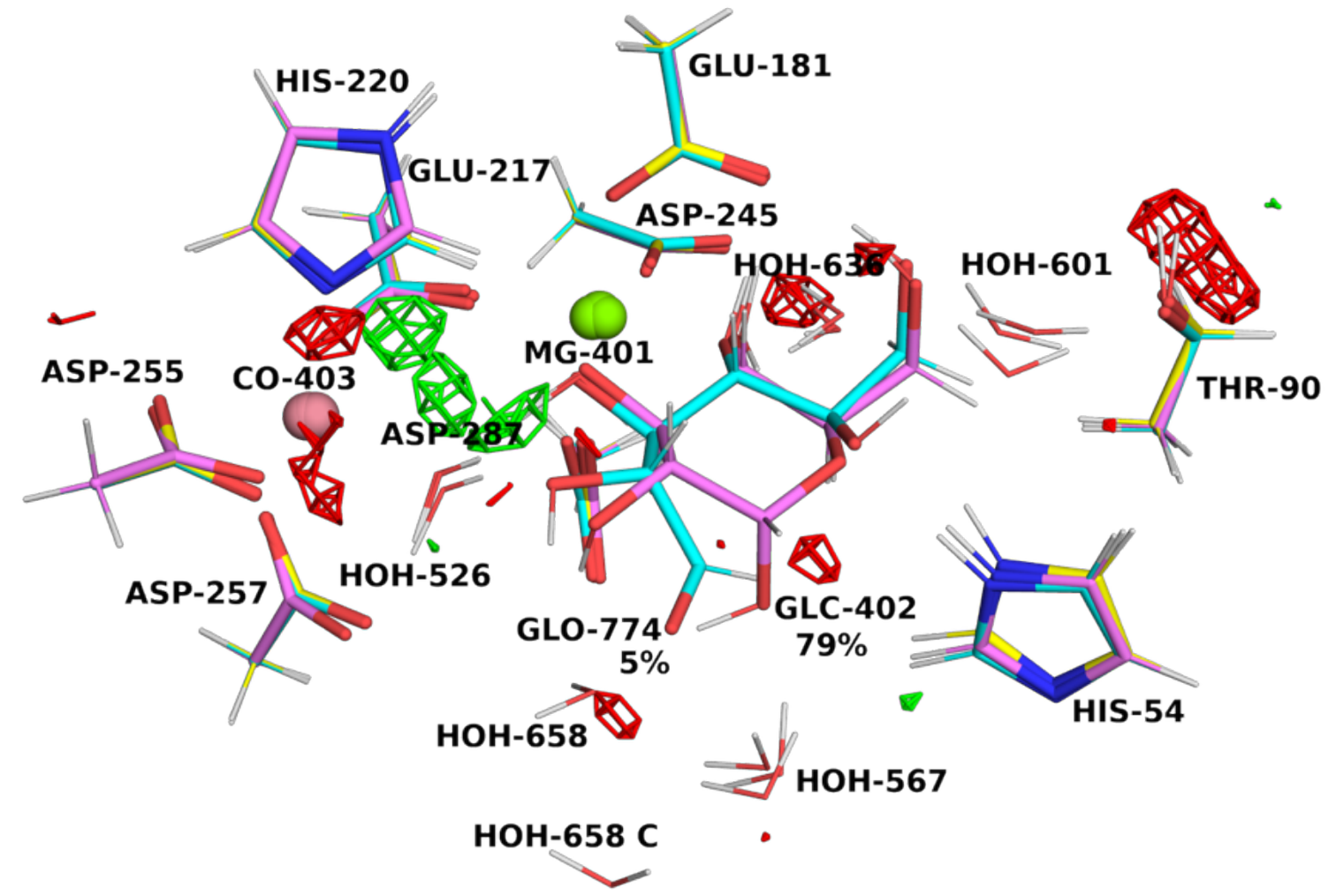
HIS-220
GLU-181
GLU-217
ASP-245
HOH-636
HOH-601
MG-401
ASP-255
CO-403
THR-90
ASP-287
HOH-526
ASP-257
GLO-774
5%
GLC-402
79%
HIS-54
HOH-658
HOH-567
HOH-658 C

d

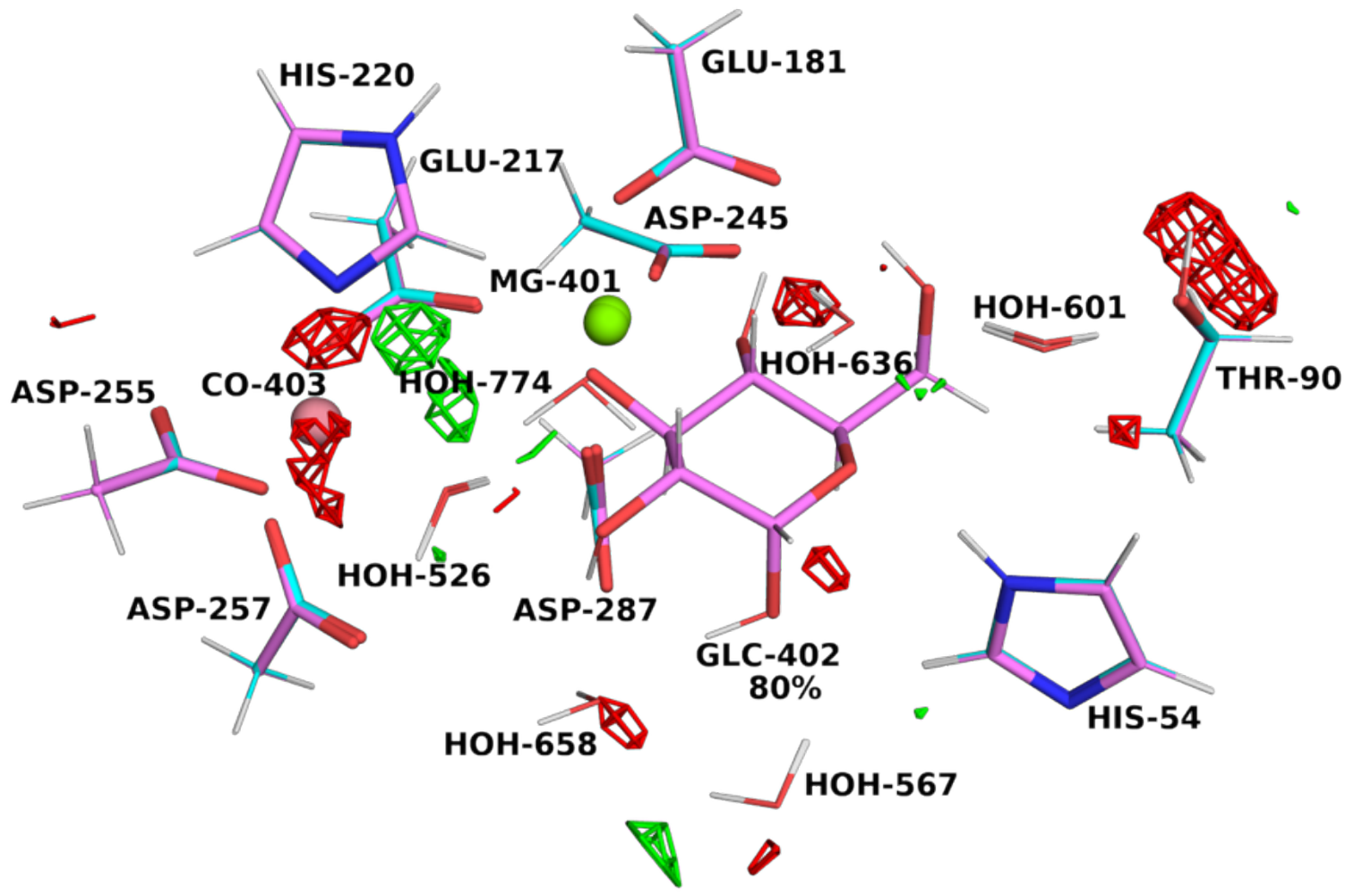
HIS-220
GLU-181
GLU-217
ASP-245
MG-401
HOH-601
HOH-636
ASP-255
CO-403
HOH-774
THR-90
HOH-526
ASP-257
ASP-287
GLC-402
80%
HIS-54
HOH-658
HOH-567

e

Figure S3: SCR (no-QM) structures for xylose isomerase (PDB ID: 6QNI) with His-54 protonated on both ND1 and NE2. a) Structure with 100% Glc. b) Structure with 79% Glc, 5% Glo and 16% no substrate. c) Structure after a movement of HOH-658 in the 16% conformation. d) Structure after deleting HOH 658 in the 5% conformation. e) Structure with 80% Glc and 20% no substrate. The conformation with Glc is depicted in pink, the one with Glo in blue and the one without substrate in yellow. The $mF_o - DF_c$ difference maps are contoured at +3σ (green) and –3σ (red).

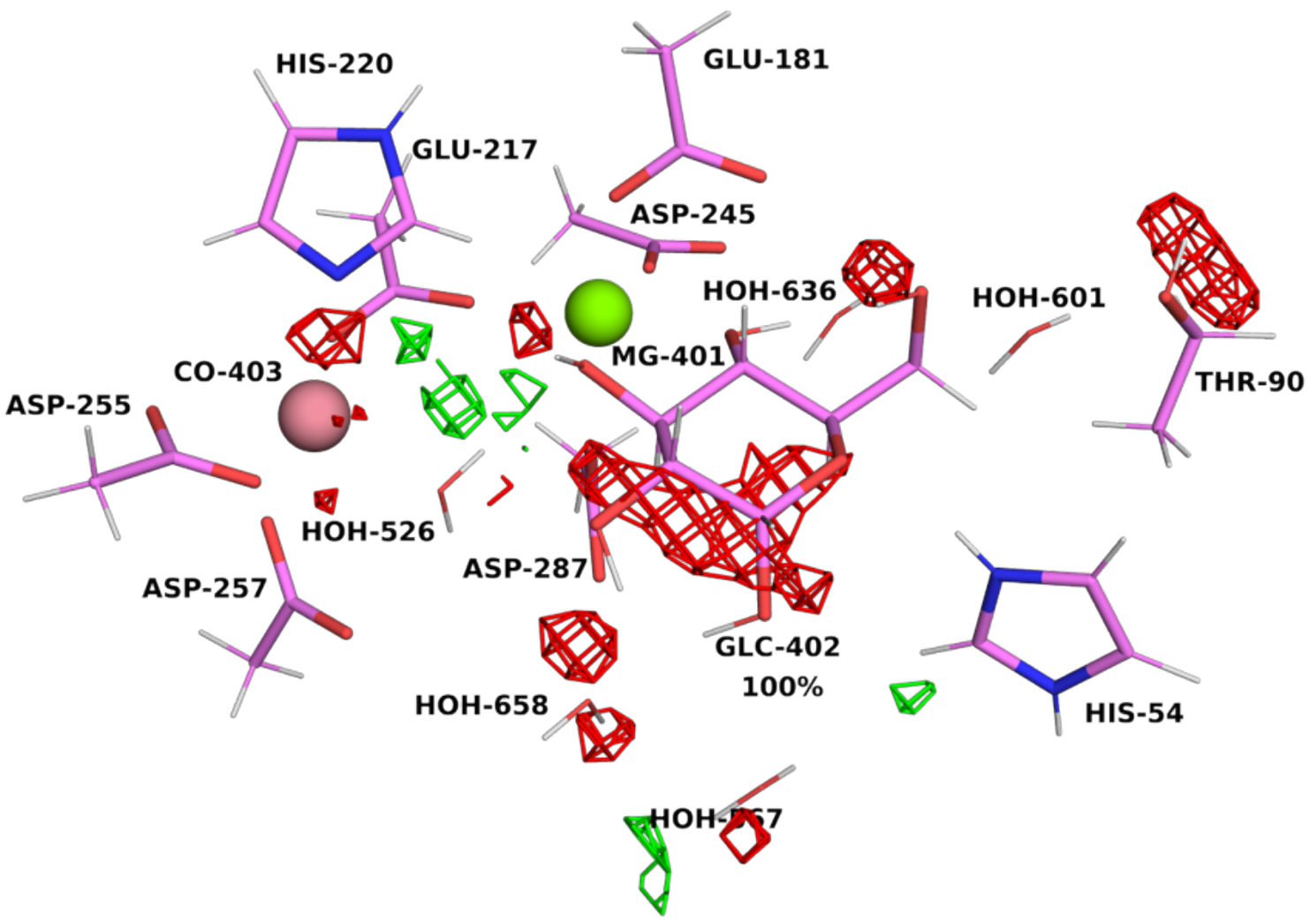

a

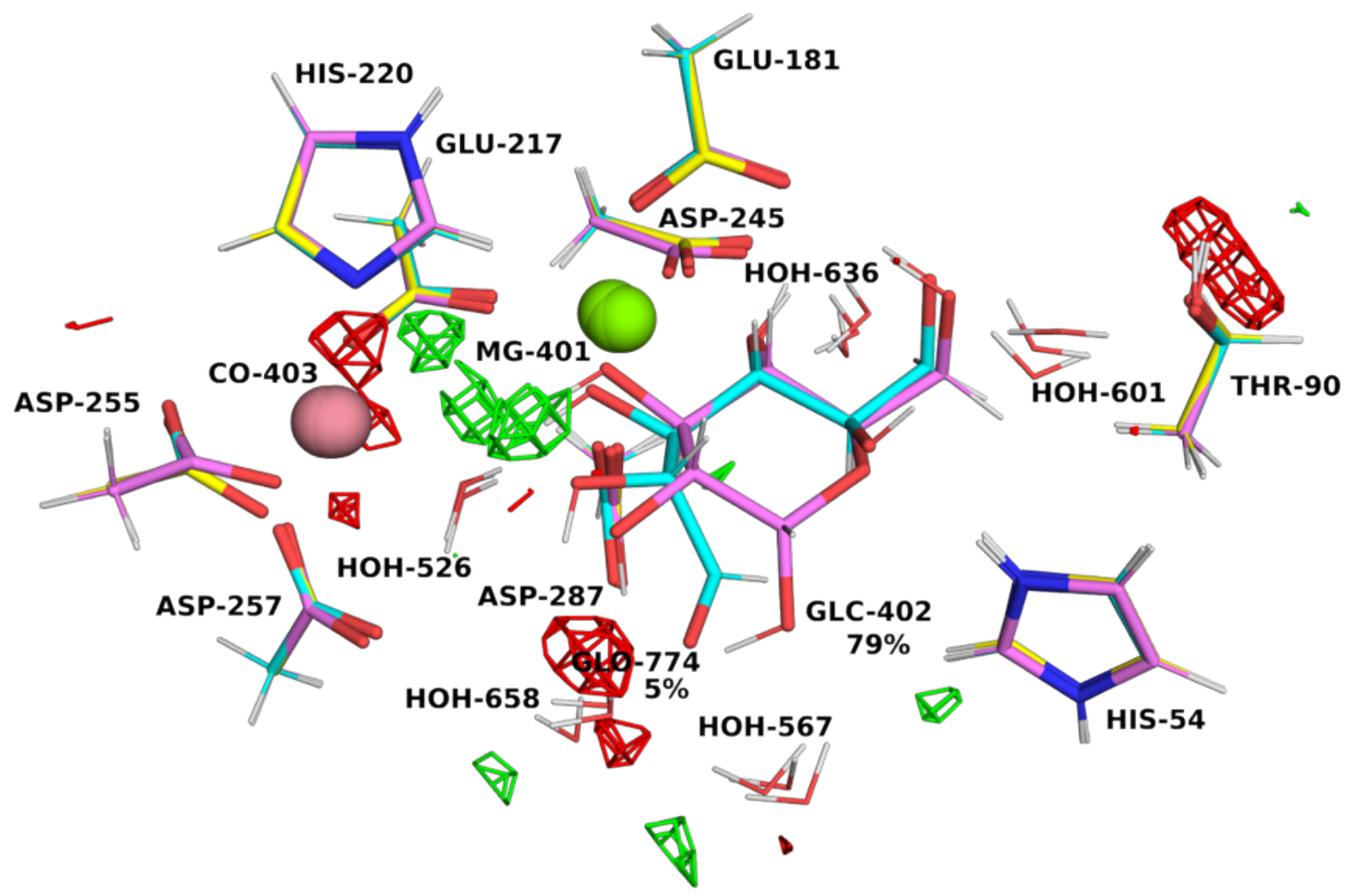

b

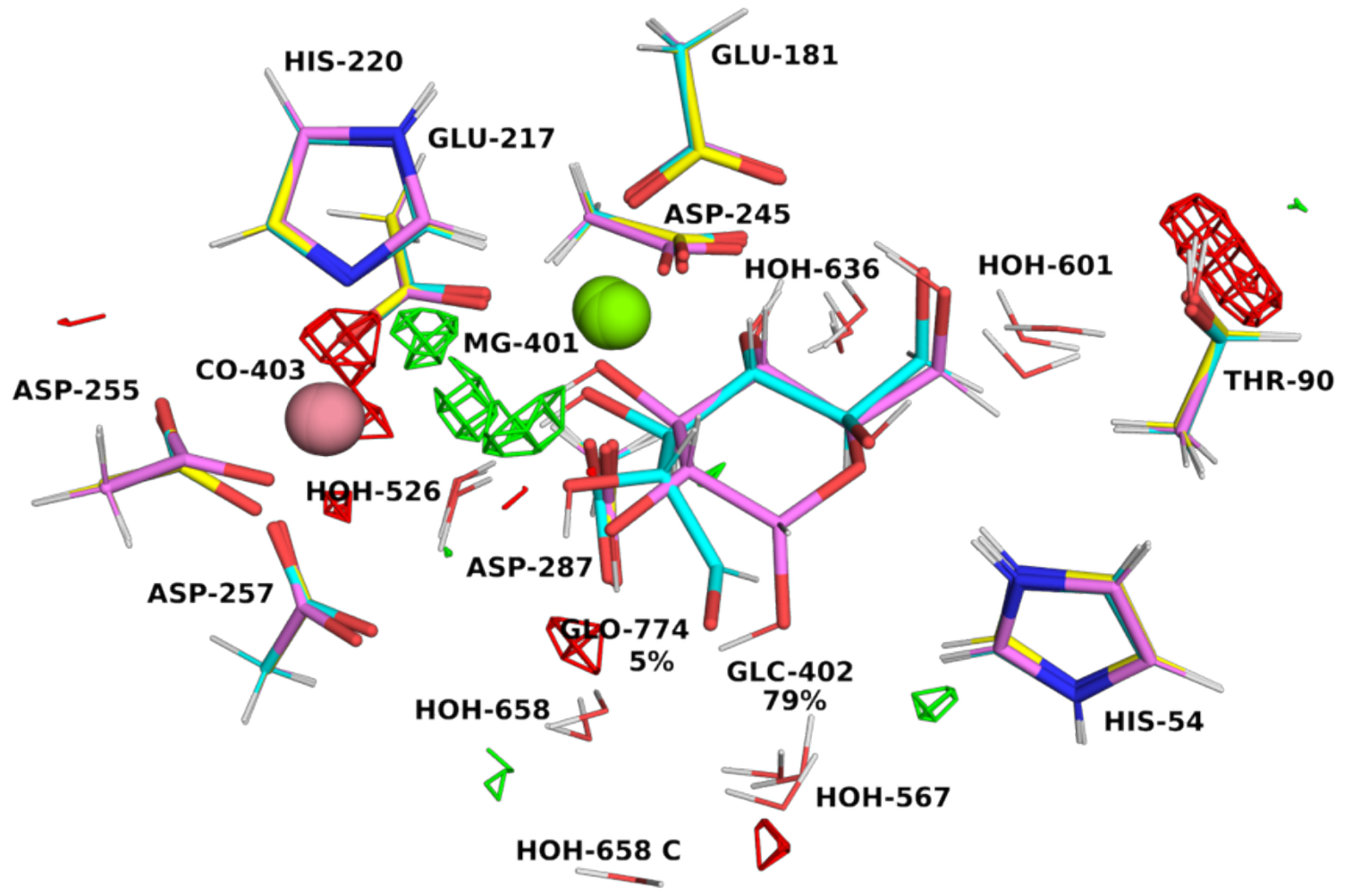
HIS-220
GLU-181
GLU-217
ASP-245
HOH-636
HOH-601
MG-401
CO-403
ASP-255
THR-90
HOH-526
ASP-287
ASP-257
GLC-774
5%
GLC-402
79%
HIS-54
HOH-658
HOH-567
HOH-658 C

c

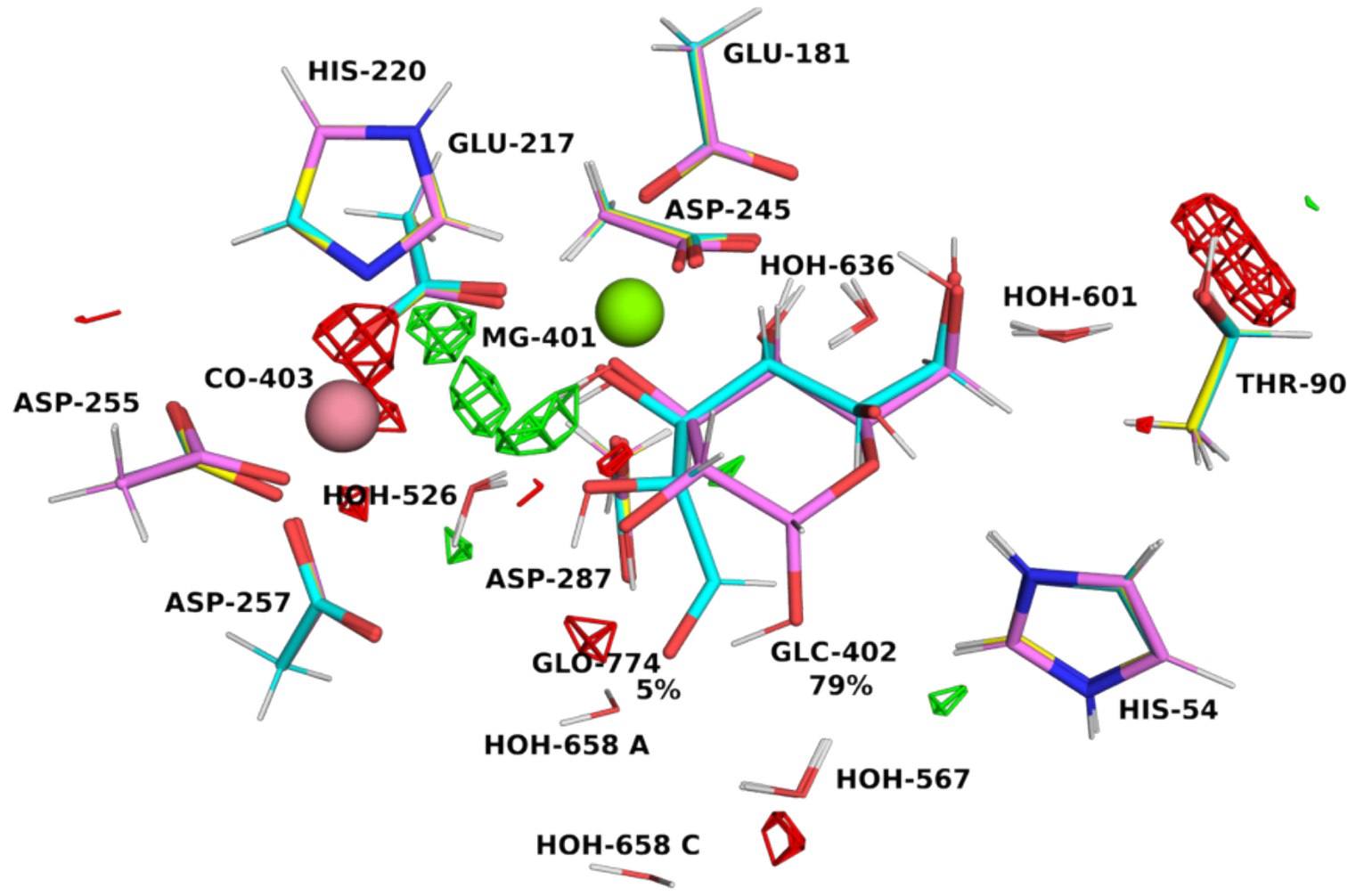
HIS-220
GLU-181
GLU-217
ASP-245
HOH-636
HOH-601
MG-401
CO-403
ASP-255
THR-90
HOH-526
ASP-287
ASP-257
GLC-774
5%
GLC-402
79%
HIS-54
HOH-658 A
HOH-567
HOH-658 C

d

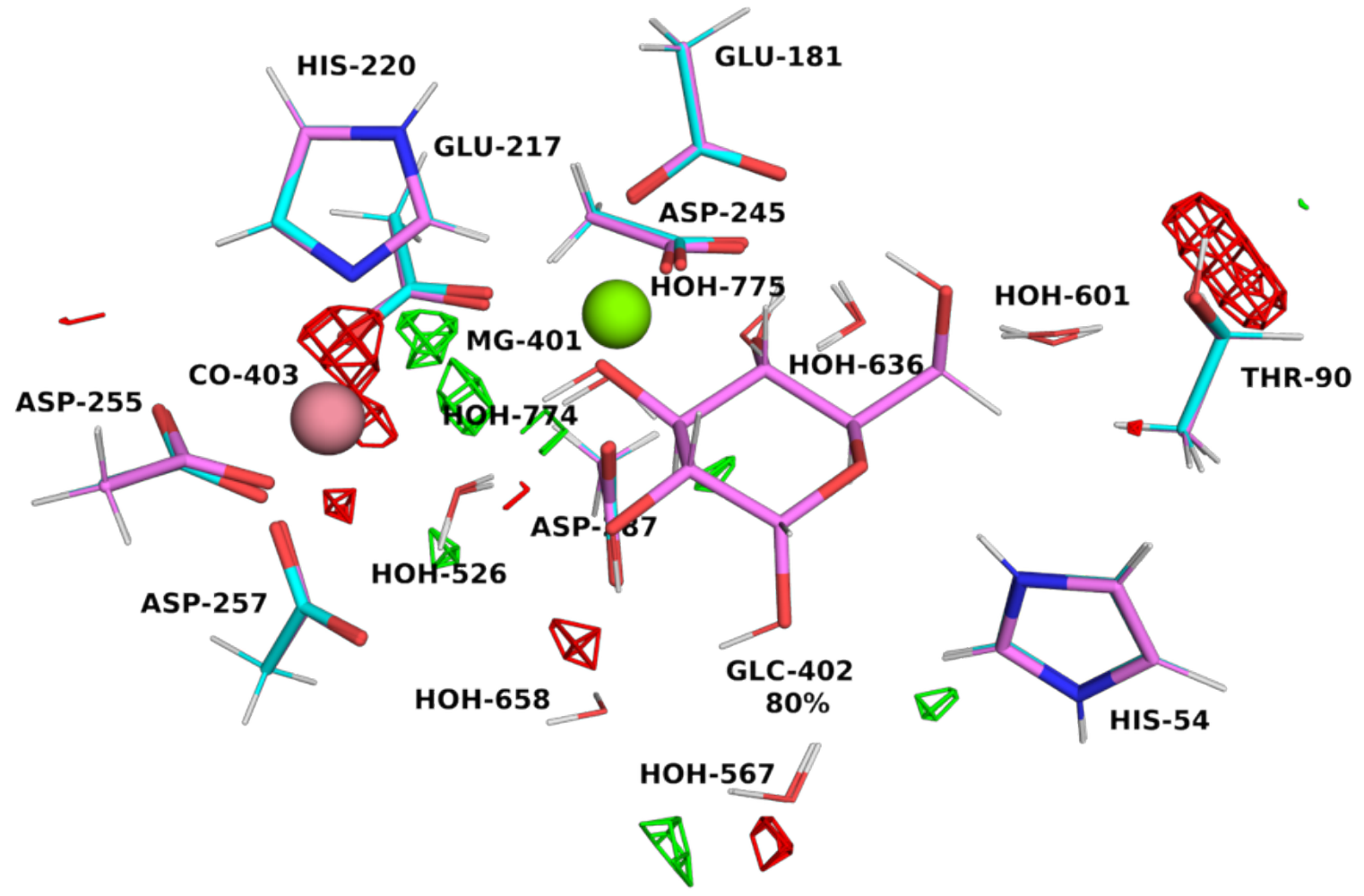
HIS-220
GLU-181
GLU-217
ASP-245
HOH-775
HOH-601
MG-401
HOH-636
CO-403
ASP-255
THR-90
HOH-774
HOH-526
ASP-257
GLC-402
80%
HOH-658
HIS-54
HOH-567

e

Figure S4. Polder maps of Asp-110 for the QR structures of the 2.3 ms state of fluoroacetate dehalogenase (PDB id 6QHP, chain A), QR1–7 in Table 1. The $mF_o - DF_c$ polder maps are contoured at +3σ (green) and –3σ (red). The different conformations are colour-coded on the C atoms in dark green, light green, magenta and brown, respectively.

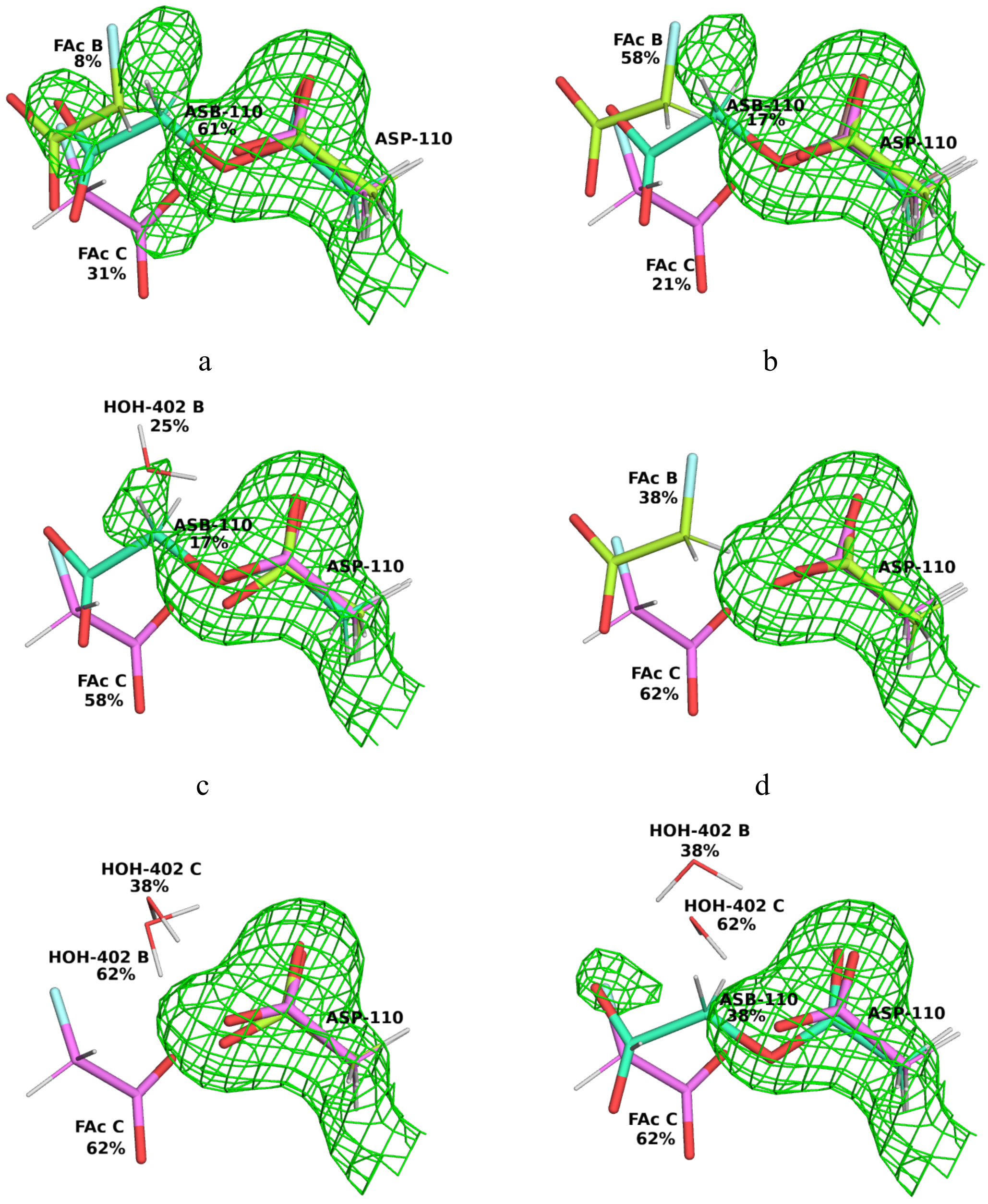

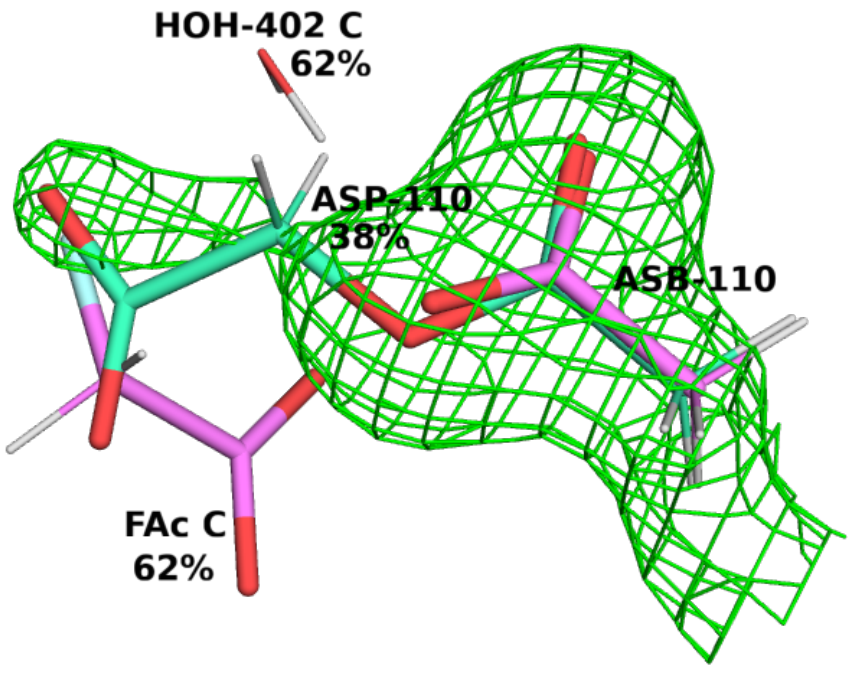

g

Figure S5: SCR (no-QM) structures of the 2.3 s state of fluoroacetate dehalogenase (PDB ID: 6QHP). All structures show the active site of the A chain and they are explained in Tables 1 and S4 (the SCR structures have the same conformations as the QR structures with the same number). The different conformations are colour-coded on the C atoms in dark green, light green, magenta and brown, respectively. The $mF_o - DF_c$ difference maps are contoured at $+3\sigma$ (green; no significant negative differences are seen in the structures).

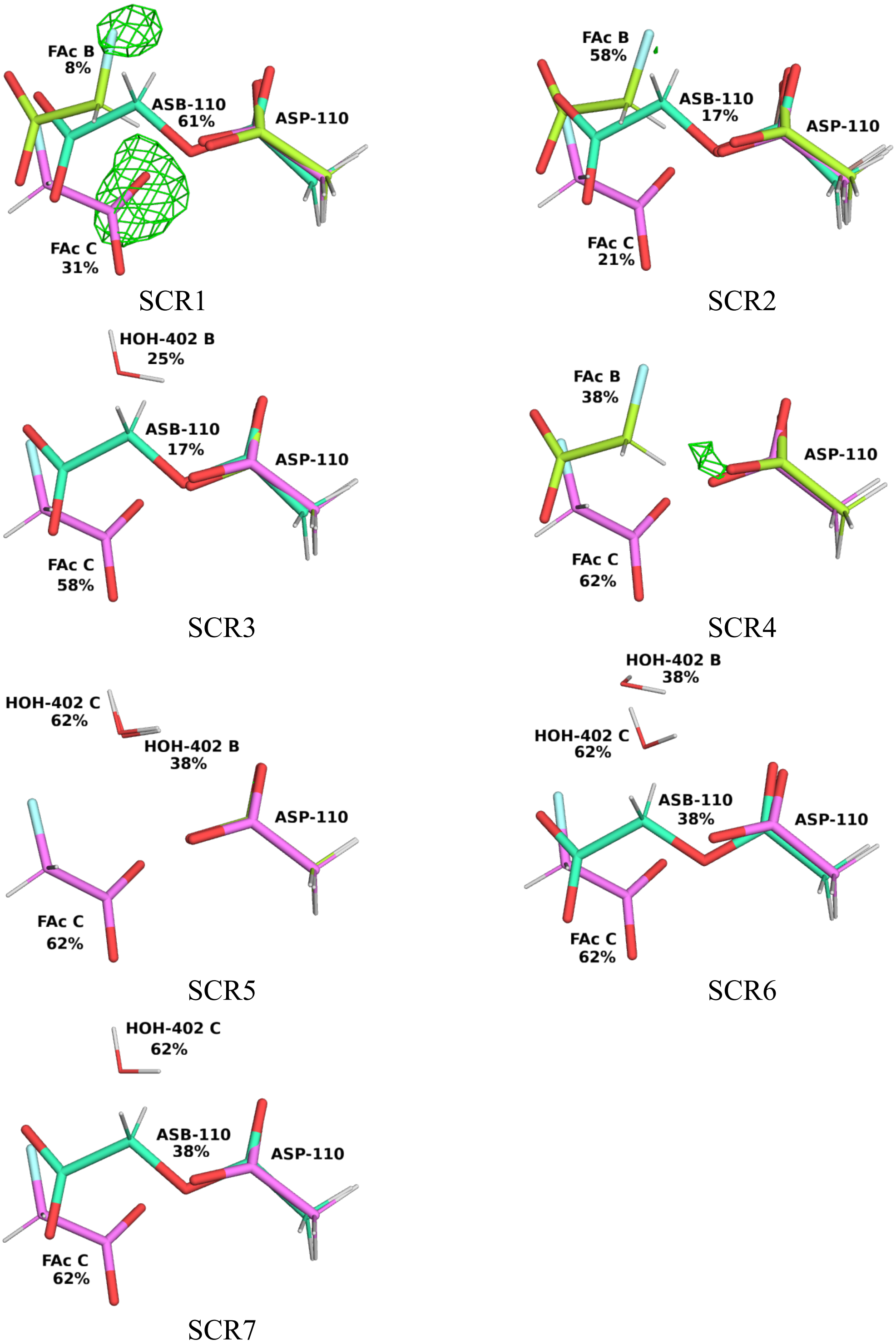

Figure S6. SCR structures (no-QM) of the 2.1 s state of fluoroacetate dehalogenase (PDB ID: 6GXT, chain A). All structures show the active site of the A chain and they are explained in Table S5 (the SCR structures have the same conformations as the QR structures with the same number, shown in Figure 5b–e). The two conformations are colour-coded on the C atoms in green and magenta, respectively. The $mF_o - DF_c$ difference maps are contoured at +3σ (green) and –3σ (red).

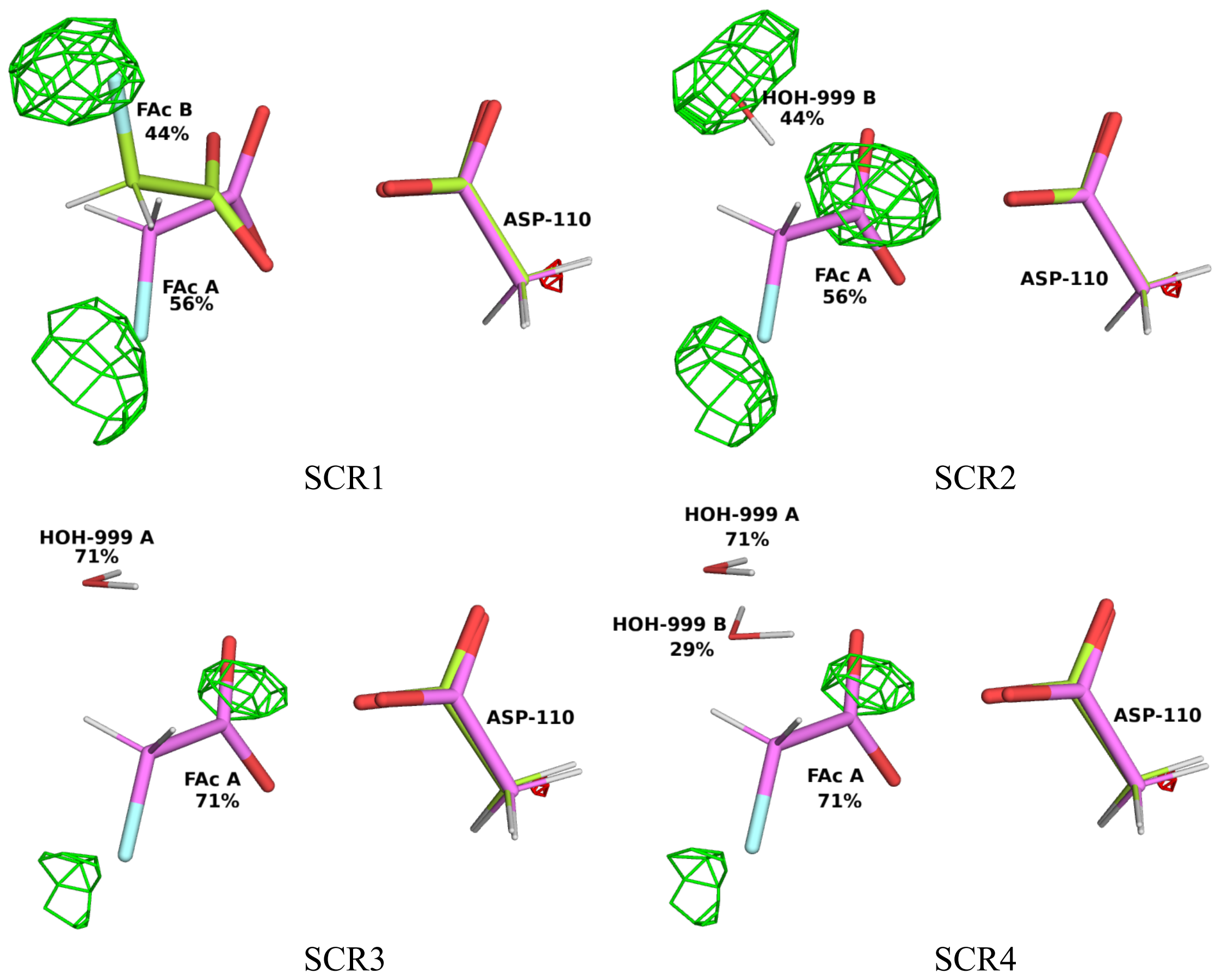

Figure S7: SCR (no-QM) structures of the 4.5 s state of fluoroacetate dehalogenase (PDB ID: 6QHW chain A). All structures show the active site of the A chain and they are explained in Table S5 (the SCR structures have the same conformations as the QR structures with the same number, shown in Figures 6b–d). The two conformations are colour-coded on the C atoms in green and magenta, respectively. The $mF_o - DF_c$ difference maps are contoured at +3σ (green) and –3σ (red).

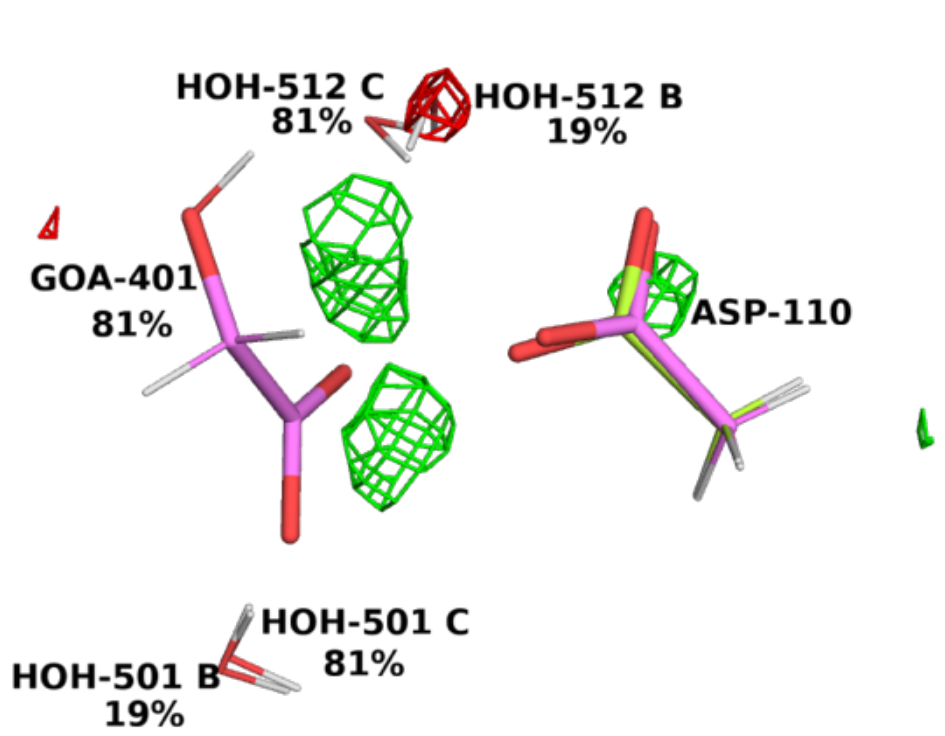

SCR1

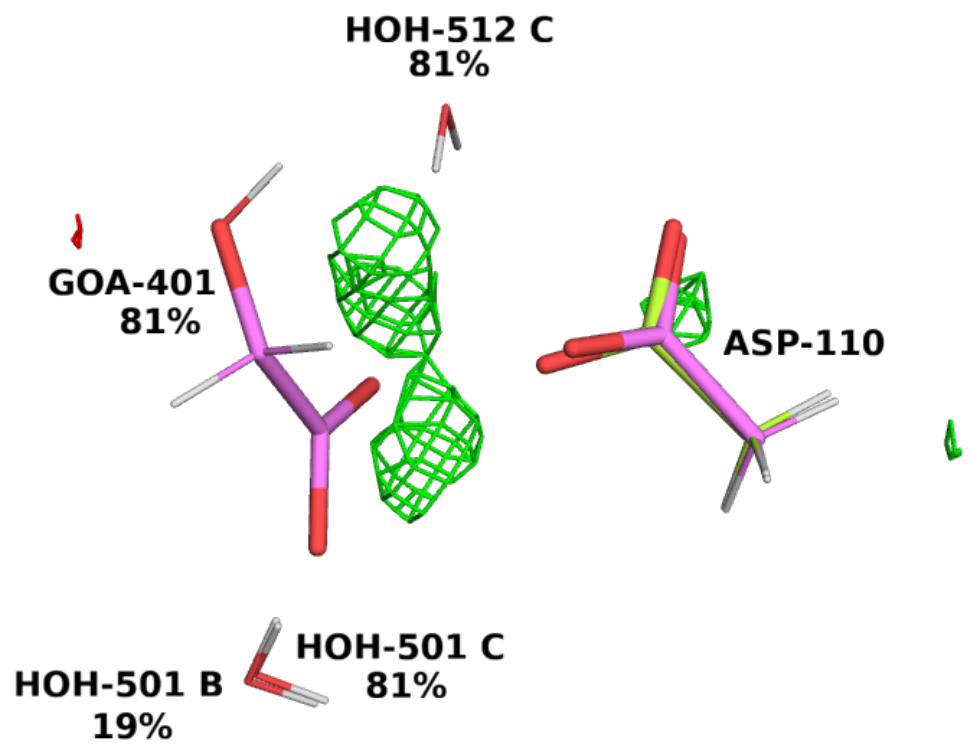

SCR2

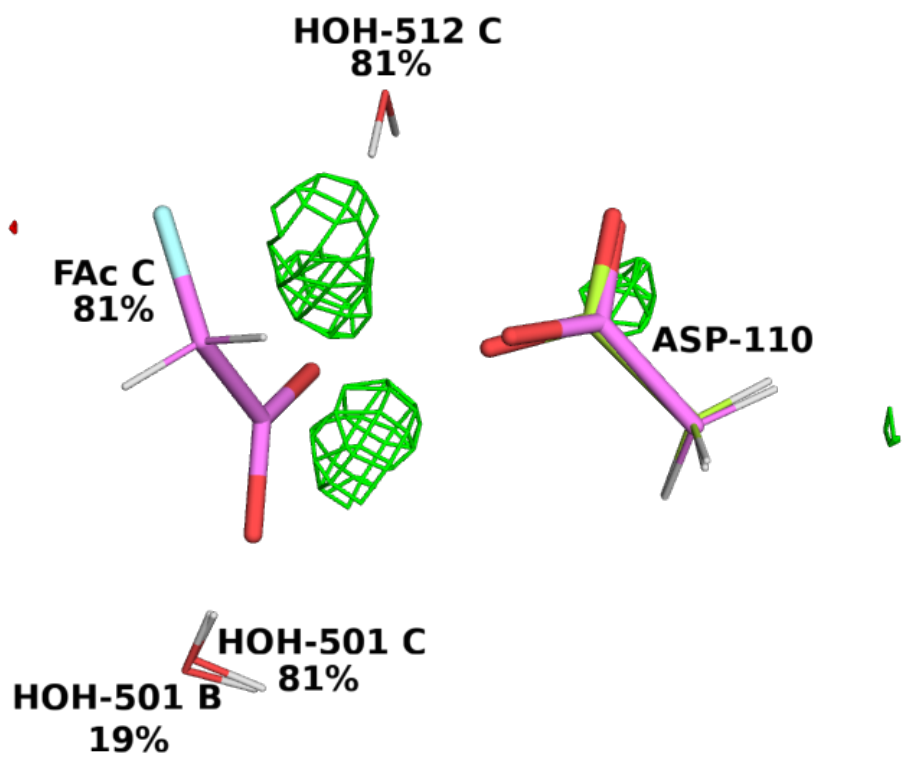

SCR3

Figure S8. Our preferred QR structures for all time-points of fluoroacetate dehalogenase, from 0 to 27 s.

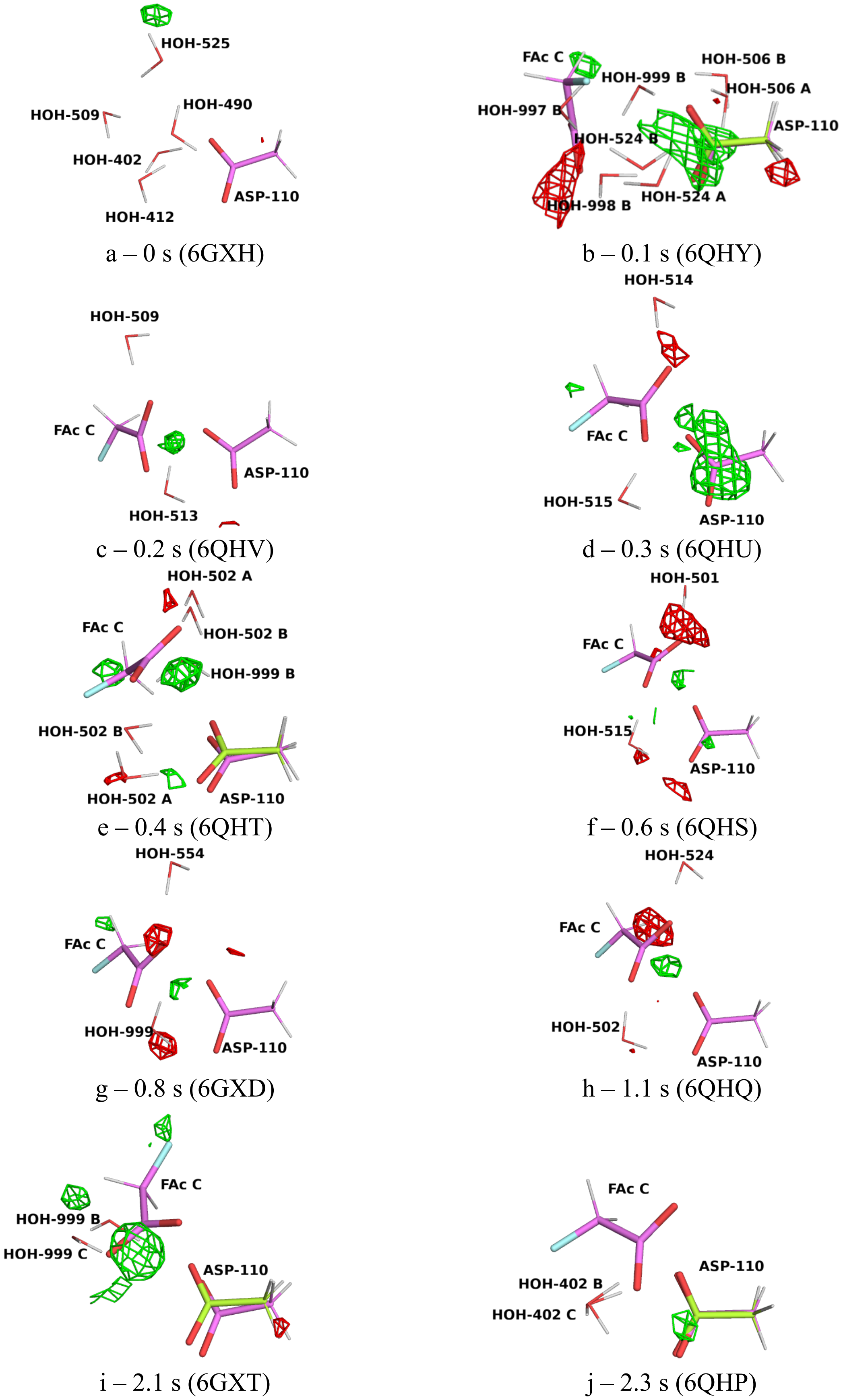

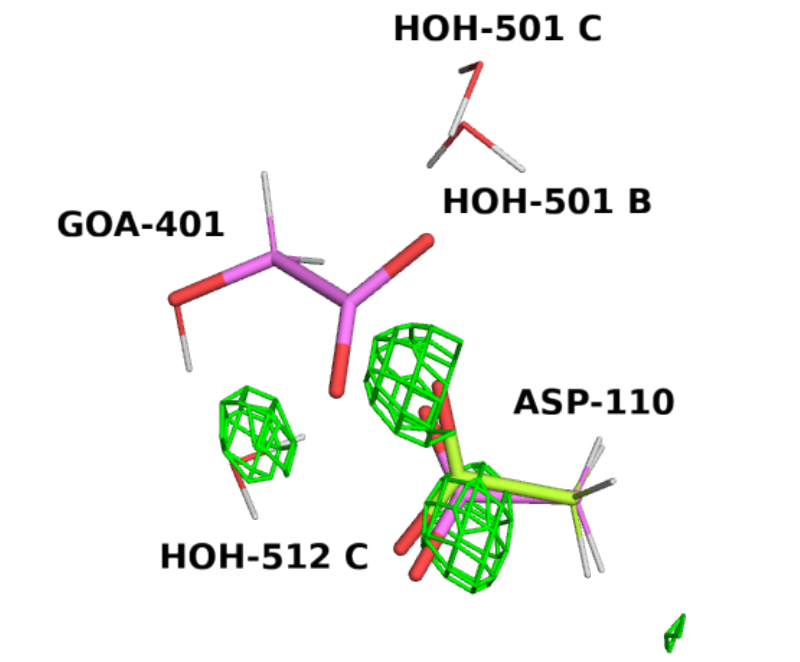

k – 4.5 s (6QHW) with GOA

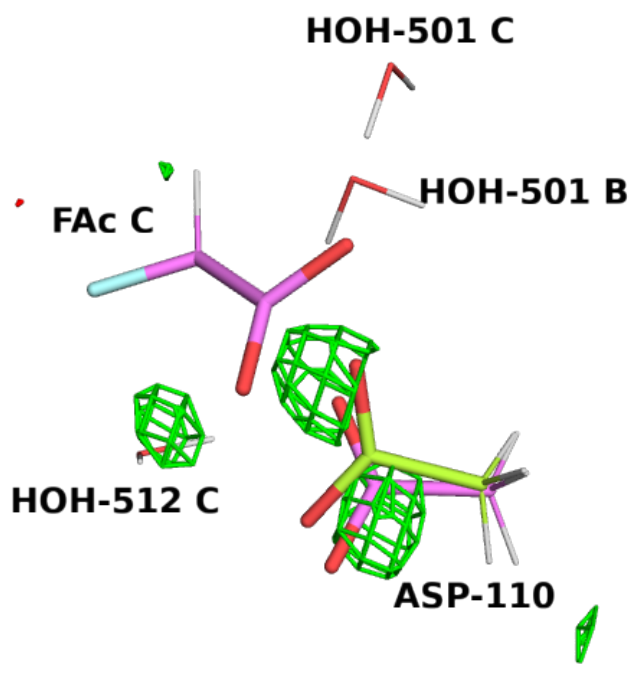

l – 4.5 s (6QHW) with FAc

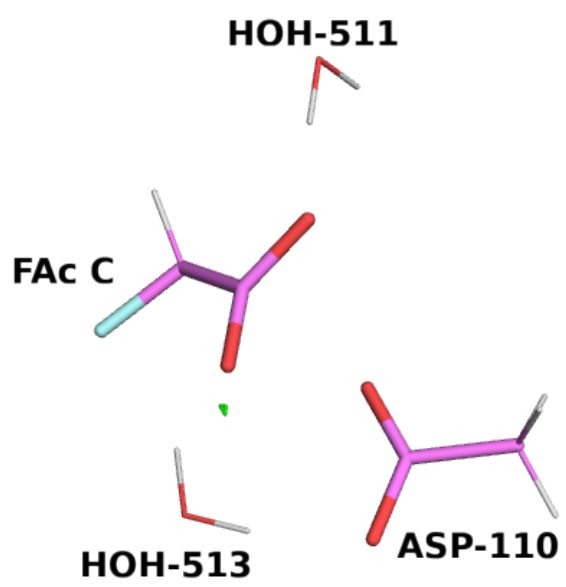

m – 7 s (6QHZ)

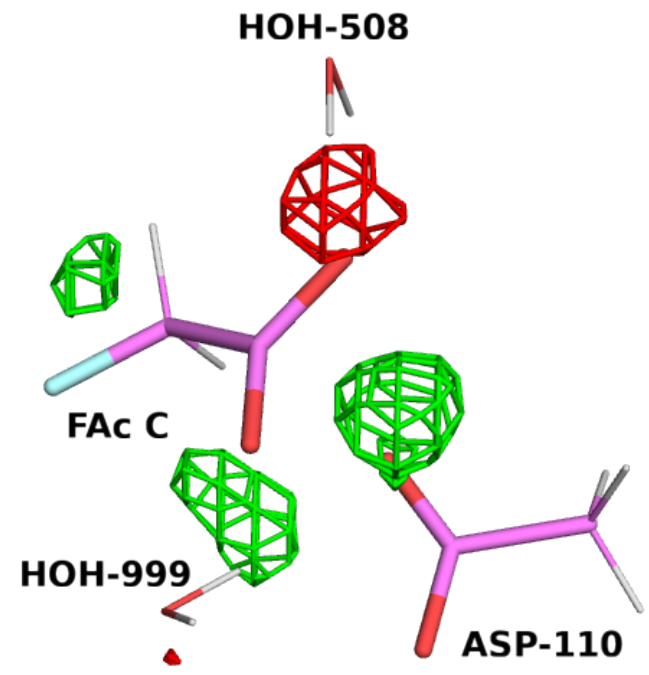

n – 9 s (6QI0)

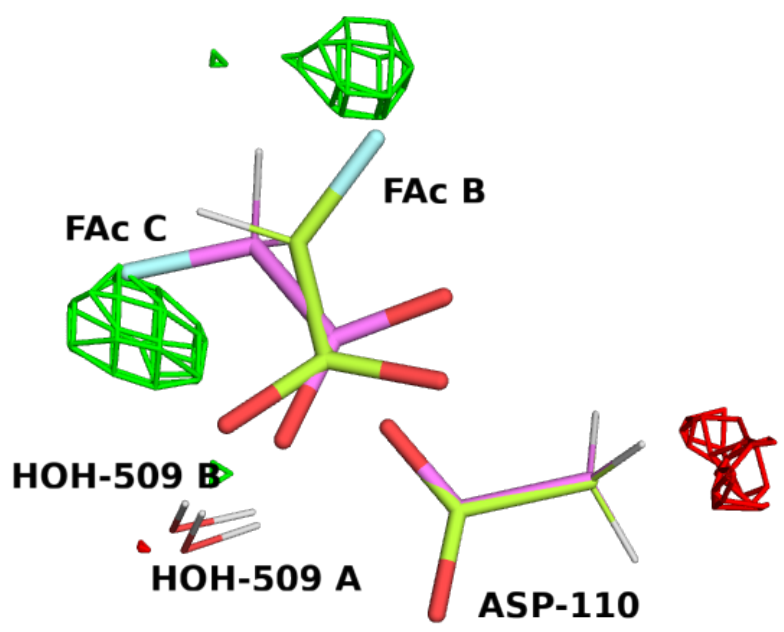

o – 13 s (6QI1)

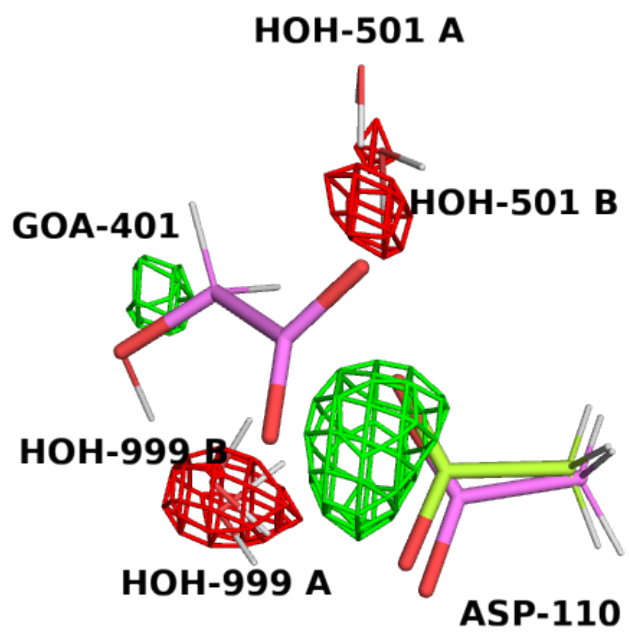

p – 14 s (6QI2) with GOA

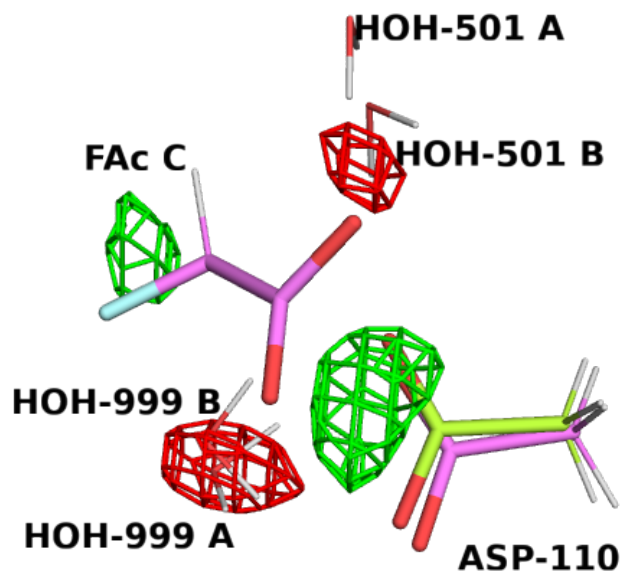

q – 14 s (6QI2) with FAc

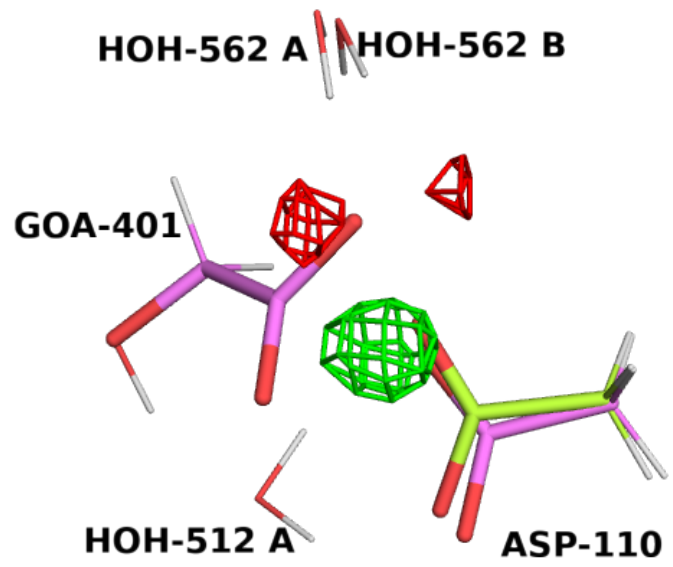

r – 27 s (6QI3) with GOA

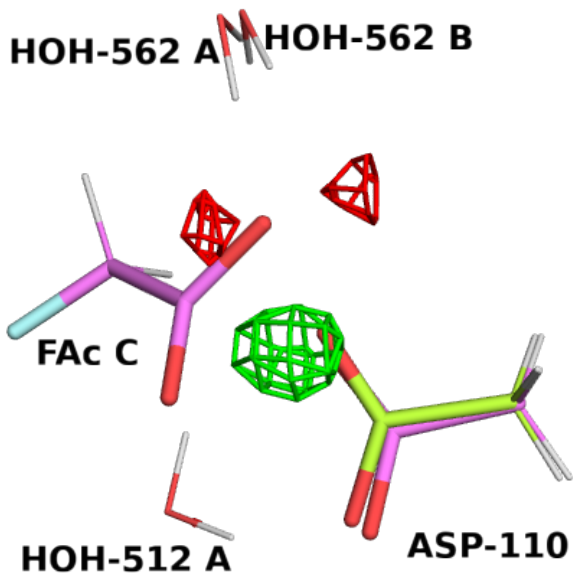

s – 27 s (6QI3) with FAc

Figure S9. Our preferred QR structures for all time-points of chain B of fluoroacetate dehalogenase, from 2.3 to 9 s.

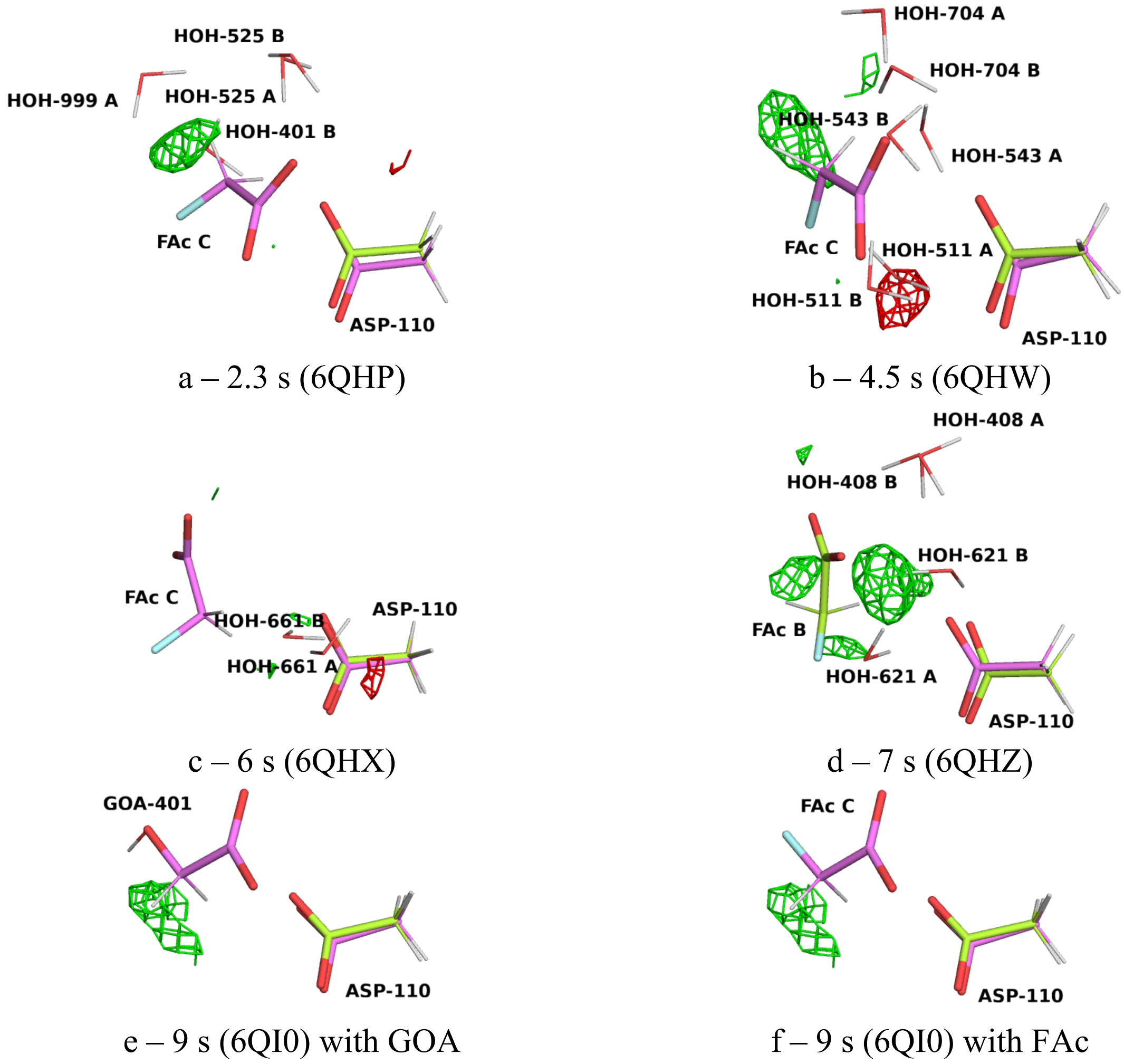

a – 2.3 s (6QHP)

b – 4.5 s (6QHW)

c – 6 s (6QHX)

d – 7 s (6QHZ)

e – 9 s (6QI0) with GOA

f – 9 s (6QI0) with FAc

Figure S10. SCR (no-QM) structures of the 7 s state of fluoroacetate dehalogenase (PDB ID: 6QHZ). All structures show the active site of the B chain and they are explained in Tables 2 and S5 (the SCR structures have the same conformations as the QR structures with the same number). The two conformations are colour-coded on the C atoms in green and magenta, respectively. The $mF_o - DF_c$ difference maps are contoured at $+3\sigma$ (green) and -3σ (red).

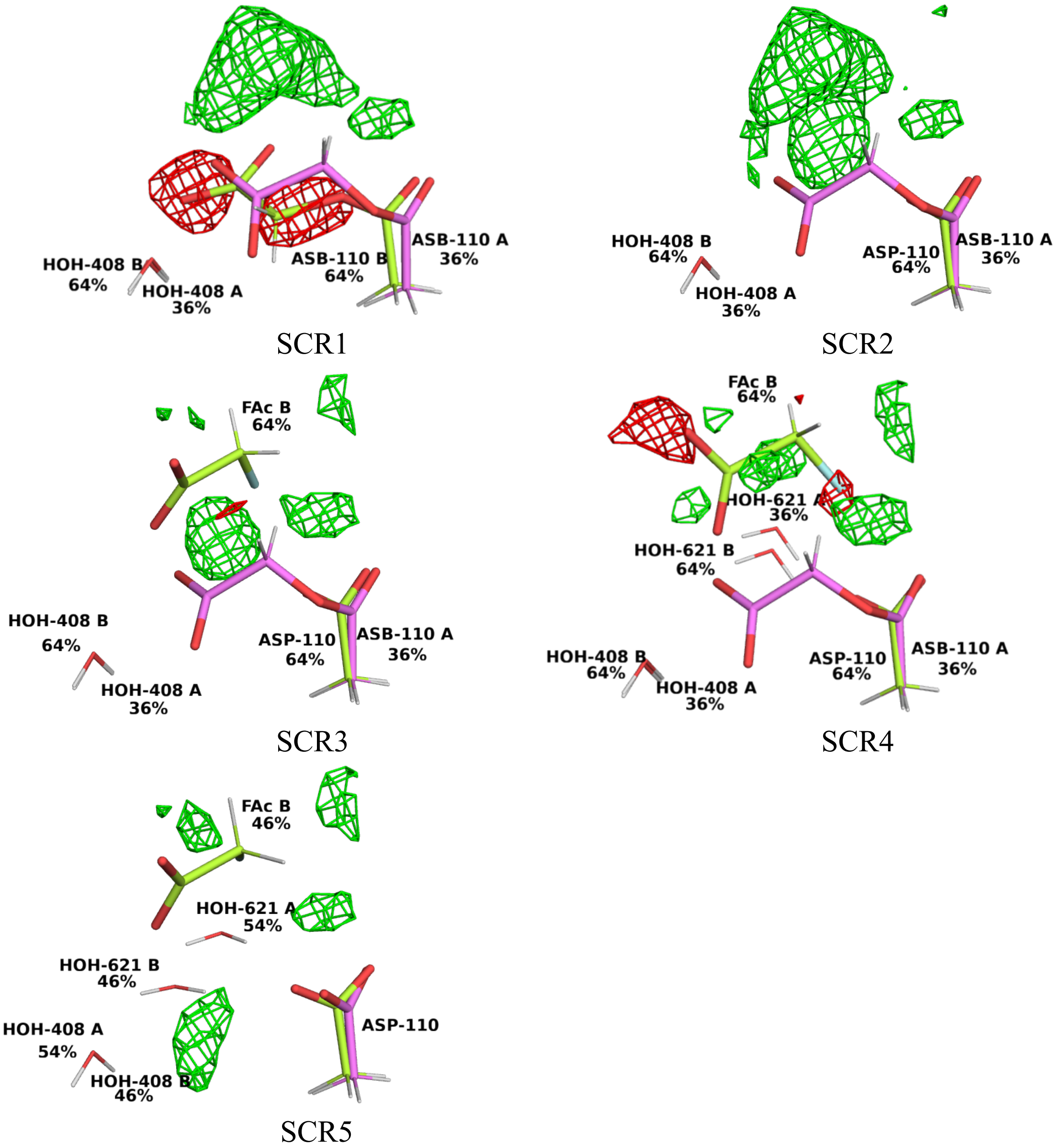

Supplement: Supplementary file 1 [file m-13-00828-sup2.pdf]
